# MicroPC (μPC): A comprehensive resource for predicting and comparing plant microRNAs

**DOI:** 10.1186/1471-2164-10-366

**Published:** 2009-08-07

**Authors:** Wuttichai Mhuantong, Duangdao Wichadakul

**Affiliations:** 1 Information Systems Laboratory, National Center for Genetic Engineering and Biotechnology (BIOTEC),113 Thailand Science Park, Phaholyothin Road, Klong 1, Klong Luang, Pathumthani, Thailand

## Abstract

**Background:**

Plant microRNA (miRNA) has an important role in controlling gene regulation in various biological processes such as cell development, signal transduction, and environmental responses. While information on plant miRNAs and their targets is widely available, accessible online plant miRNA resources are limited; most of them are intended for economically important crops or plant model organisms. With abundant sequence data of numerous plants in public databases such as NCBI and PlantGDB, the identification of their miRNAs and targets would benefit researchers as a central resource for the comparative studies of plant miRNAs.

**Results:**

MicroPC (μPC) is an online plant miRNA resource resulted from large-scale Expressed Sequence Tag (EST) analysis. It consists of 4,006 potential miRNA candidates in 128 families of 125 plant species and 2,995 proteins (4,953 EST sequences) potentially targeted by 78 families of miRNA candidates. In addition, it is incorporated with 1,727 previously reported plant mature miRNA sequences from miRBase. The μPC enables users to compare stored mature or precursor miRNAs and user-supplied sequences among plant species. The search utility allows users to investigate the predicted miRNAs and miRNA targets in detail via various search options such as miRNA family and plant species. To enhance the database usage, the prediction utility provides interactive steps for determining a miRNA or miRNA targets from an input nucleotide sequence and links the prediction results to their homologs in the μPC.

**Conclusion:**

The μPC constitutes the first online resource that enables users to comprehensively compare and predict plant miRNAs and their targets. It imparts a basis for further research on revealing miRNA conservation, function, and evolution across plant species and classification. The μPC is available at http://www.biotec.or.th/isl/micropc.

## Background

MicroRNA (miRNA) is a class of non-coding small RNAs with a single strand molecule of 18–22 nucleotides. It plays a pivotal role in gene regulation in various species by targeting mRNAs at the post-transcriptional levels [1]. In plants, miRNA involves in organ development and environmental responses [2-4].

Despite accumulating studies in the identification of plant miRNAs and their targets based on both computational [5-12] and experimental [13-16] approaches, existing public resources focusing on plant miRNAs and their targets are limited to economically important crops or specially implemented for plant model organisms such as *Arabidopsis*, rice, and maize. Among them, miRU [17] is a web server for plant miRNA target prediction utilizing the concepts of nearly perfect binding between a miRNA and its targets. Plant MPSS (Massively Parallel Signature Sequencing) databases [18] of *Arabidopsis*, rice, grape, and rice blast fungus (*Magnaporthe grisea*) were developed to provide an online resource for mRNA and small RNA analyses. ASRP (*Arabidopsis *Small RNA Project) [19,20] provides a repository with tools for analysis and visualization of small RNA sequences cloned from various ecotypes and tissues of *Arabidopsis*. CSRDB (Cereal Small RNA Database) [21] consists of maize and rice small RNA sequences generated by high-throughput pyrosequencing with the provided maps to the available rice and maize genomes.

Here, we developed MicroPC (μPC) as a comprehensive online resource for predicting and comparing plant miRNAs and their targets. The μPC is resulted from applying previous steps and criteria [7,22-25] with known plant mature miRNA sequences from miRBase [26,27] and large-scale EST sequences from PlantGDB [28,29]. The μPC database consists of 4,006 predicted miRNA candidates in 128 families of 125 plant species and 2,995 proteins (4,953 EST sequences) potentially targeted by 78 families of potential miRNA candidates. It characterizes the predicted miRNAs and their targets according to plant classification (e.g., monocots, dicots, mosses) and incorporated with previously discovered 1,727 plant mature miRNAs from miRBase. With stored data, it offers users a comparative and integrative view of previously reported miRNAs and our predicted miRNA candidates among various plant species. The sequence alignment feature enables users to explore the relation and evolution of either mature or precursor miRNA sequences within a family across plant species. Moreover, the search utility provides various search options for users to explore the predicted miRNA candidates and their potential targets in detail. The prediction utility helps to predict potential miRNA candidates for an input nucleotide sequence and to scan for EST sequences of a plant species that might be targeted by an input mature miRNA sequence.

## Construction and content

### Data acquisition

We obtained 1,727 (1,022 with experiments and 705 without experiments) known mature miRNA sequences in 418 families of 21 plant species from miRBase release 12.0 (September 2008) [see Additional file 1]. To prepare and filter source sequences, we used UNAFold [30] to predict the secondary structures of the obtained non-experimental stem-loop sequences and applied the criteria in [25] to remove structural sequences which have (1) the number of mismatches between miRNA and miRNA* greater than 4, and/or (2) the number of asymmetric bulges greater than 1, and/or (3) the size of the asymmetric bulge greater than 2 [see excluded sequences in Additional file 2]. We then manually curated remaining 513 sequences and deployed them together with all sequences with experiments as source sequences for miRNA prediction. All 5,306,503 EST sequences of 173 plant species were downloaded from PlantGDB in November, 2008 [see Additional file 3]. We compiled 1,372 RNA families from Rfam 9.1 (January, 2009) [31] to filter out non-coding RNAs (ncRNAs) that are not miRNAs in the step of miRNA prediction. For EST functional annotation, the UniProtKB/Swiss-Prot and UniProtKB/TrEMBL protein data sets release 14.9 were obtained from UniProt [32]. These data sets will be updated periodically when their new releases are available.

### Prediction of potential miRNA candidates

**Figure 1:**
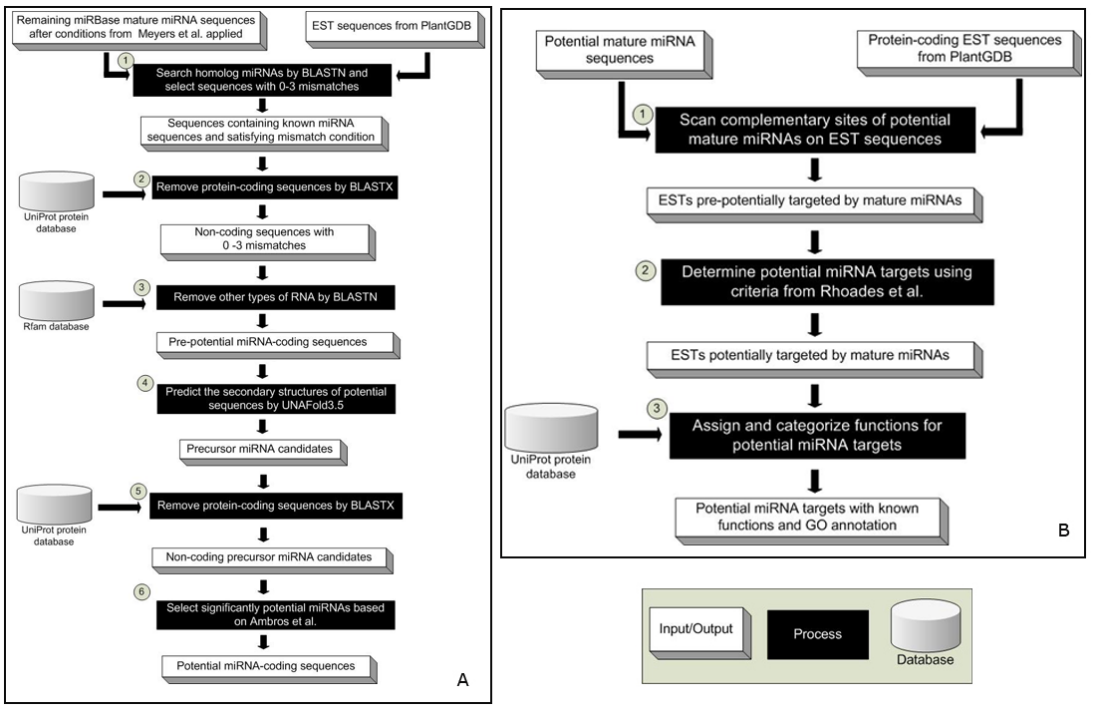
**Prediction steps of potential (A) miRNA candidates and (B) miRNA targets for plants**.

The prediction of plant miRNA candidates was primarily inferred from the study of Zhang et al. [7], consisting of six main steps (Figure 1A). First, known mature miRNA sequences from miRBase were searched against EST sequences of 173 plant species from PlantGDB using BLASTN [33] version 2.2.18 (with E-value < 100 and window size = 4) for identifying homologs. Second, EST sequences containing homolog mature miRNA sequences with the number of mismatches fewer than 4 were searched against UniProt protein database using BLASTX (with E-value < e-20) for removing protein-coding sequences. EST sequences that did not significantly hit any proteins in UniProt from the second step would be further searched against Rfam database using BLASTN (with E-value < e-8) for removing homologs of non-coding RNAs other than miRNAs. Fourth, remaining EST sequences were predicted for the secondary structure of primary miRNA (pri-miRNA) and precursor miRNA (pre-miRNA) by UNAFold which resulted in RNA secondary structures ranked by their minimal free energies (MFEs). Next, predicted precursor miRNA sequences were searched against UniProt protein database using BLASTX (with E-value < e-20) for removing possible protein-coding sequences. An EST sequence will be determined as a miRNA gene if its pre-miRNA structures with lowest MFE satisfy the criteria proposed by Ambros et al. [23]: (1) the mature miRNA sequence has to be within one arm of the hairpin secondary structure, (2) the number of mismatches between mature miRNA sequence and its miRNA* sequence is fewer than 6, and (3) there is no muti-loop in pre-miRNA hairpin secondary structure. The total 1,153 EST sequences were predicted as potential miRNA candidates in 128 families of 125 plant species. These sequences with their secondary structures, MFEs, source mature miRNA sequences from miRBase, and other prediction detailed results are collected in the μPC database.

### Prediction of ESTs targeted by miRNA candidates

The prediction of plant miRNA targets is a straightforward process since plant mature miRNAs can bind with specific mRNA targets through perfect or nearly-perfect complementarities [13,34]. Sequences of potential miRNA candidates were scanned for complementary sites on EST sequences that significantly hit some proteins in UniProt database based on BLASTX (E-value < e-20). An EST sequence would be considered as a potential target if it passes the criteria used in Rhoades et al. [22,35]: (1) the number of mismatches (including G:U pair [36]) between sequence of potential miRNA candidate and its complementary site on the EST is fewer than 4, and (2) there is no gap in the complementary site. As specific positions of mismatches affect miRNA targeting [37,38], we also collected these positions to enable the query of predicted targets with specific mismatch positions of interest. Total 2,995 proteins (4,953 EST sequences) potentially targeted by 78 families of miRNA candidates were assigned with Gene Ontology (GO) [39], categorized by GO ID (e.g., GO:0000166) and GO term (e.g., nucleotide binding), and stored in the μPC database. Figure 1B shows the procedure of potential miRNA target prediction.

### Prediction accuracy and coverage

To assess the prediction accuracy and coverage, 936 experimental precursor miRNA sequences from miRBase were used as input for the miRNA prediction procedure and 761 out of 936 (81.3%) were correctly predicted as miRNA genes [see Additional file 4]. Other sequences were mostly ruled out during the secondary structure prediction. With one thousand repeats of 936 randomly generated sequences, the average number of random sequences predicted as miRNA genes is extremely low (0.00064%). To assess whether variable source mature miRNA sequences affect prediction results, we applied the same prediction steps to the 936 precursor miRNA sequences with leave-one-out of source mature miRNA sequences. The removal of common miRNAs or miRNAs with several members in a family has little or no effects on prediction results. In contrast, a removed species-specific miRNA sequence such as miRNA 1063 in moss will reduce the number of prediction coverage [see Additional files 5 and 6]. These measurements suggest that our miRNA prediction procedure could predict potential miRNA candidates with confidence and has limitation in coverage due to homology-based approach.

## Utility and discussion

The μPC offers three main interactive pages for comparing, searching, and predicting plant miRNAs and their targets as described below.

### Comparative miRNAs

**Figure 2:**
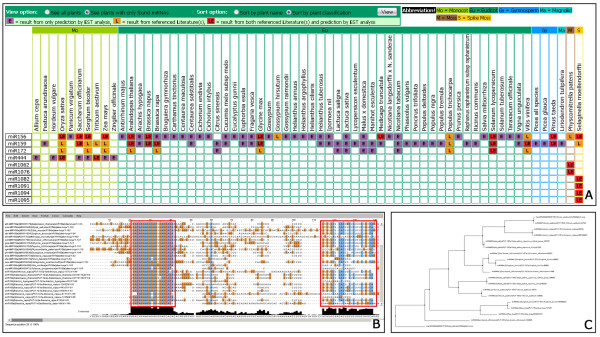
**Comparative miRNAs**. (A) a comparative matrix of miRNA 156, miRNA 159, miRNA 172, miRNA 444, miRNA 1062, miRNA 1076, miRNA 1082, miRNA 1091, miRNA 1094, miRNA 1095, and miRNA 1098 sorted by plant classification (e.g., monocots, eudicots) and filtered for only plants found with the query, (B) a multiple sequence alignment by CLUSTALW via Jalview of precursor miRNA sequences from both miRBase and predicted EST sequences of miRNA 156 family, two red boxes are the highly conserved mature miRNA and miRNA* sequences among miRNA members, (C) a phylogenetic tree of miRNA 444 from monocots.

The comparative utility enables users to explore the conservation of known and predicted miRNAs among plant species in integrative views. Users may select miRNAs of interest and submit for their availability among plant species. Cells with different colors in a matrix view of selected miRNAs and plants represent available miRNAs from three sources: our EST analysis, all previous reports from miRBase, or both. Figure 2A reveals the conservation of miRNA 156, miRNA 159, miRNA 172 across plant groups, while miRNA 444 seems to be monocot-specific. miRNA 1062 and miRNA 1076 appear to be moss-specific and miRNA 1082, miRNA 1091, miRNA 1094, miRNA 1095, and miRNA 1098 seem to be spike moss-specific. Besides the comparative matrix view, users may explore the multiple sequence alignment and evolution of either mature or precursor miRNA sequences from miRBase, our prediction results, and user-supplied sequences via incorporated CLUSTALW [40] and Jalview [41] (Figures 2B and 2C).

### Search

**Figure 3:**
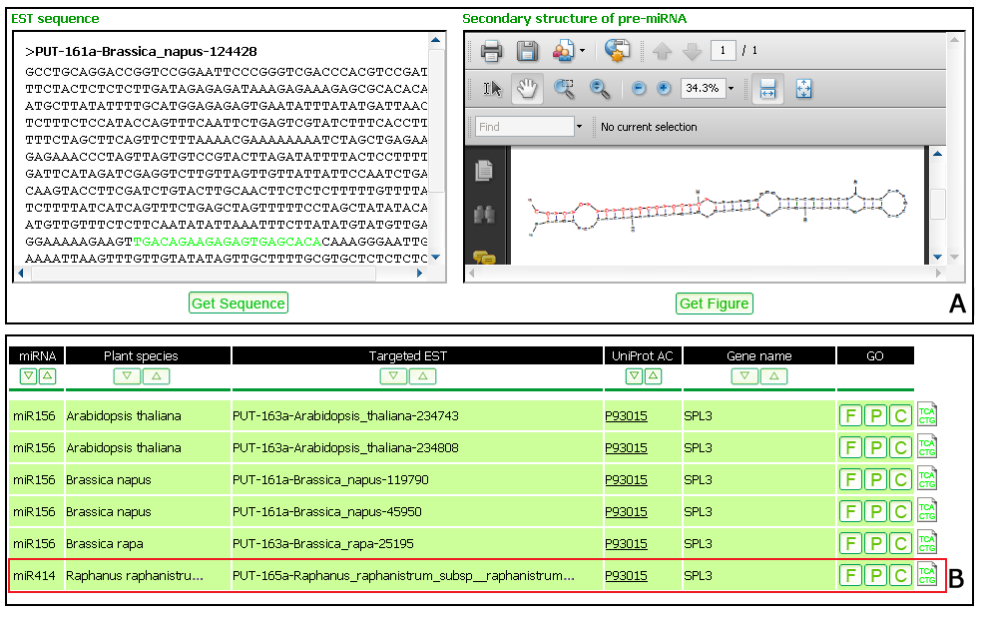
**Search results of a miRNA and miRNA targets**. (A) an EST sequence of *Brassica napus *and its secondary structure predicted as a miRNA 156 gene, (B) targeted EST sequences from all plants searched by UniProt accession number [Swiss-Prot:P93015] (SPL3 protein in *Arabidopsis*), the red box suggests that targeted EST sequences in *Raphanus raphanistrum *could be also the targets of miRNA 414 besides miRNA 156.

The search utility provides various ways for users to access predicted miRNA candidates and potential miRNA targets in detail. Users may search for predicted miRNAs by miRNA family (e.g., miR156), plant species (e.g., *Arabidopsis thaliana*), mature miRNA sequence (e.g., ugacagaagagagugagcac), and miRBase AC or ID (e.g., MIMAT0000166 or ath-MIR156a). The miRNA search result mainly includes a list of EST accession numbers potentially encoding miRNAs, their referenced mature miRNA sequences from miRBase, and a link to detailed prediction results such as the secondary structure of pre-miRNA sequence, mature miRNA sequence, positions, number of mismatches, GC content, and MFEI (minimal folding free energy index) [42]. Users could also export the EST source sequence and its secondary structure of pre-miRNA for a specific record (Figure 3A). Users may search for ESTs potentially targeted by miRNA candidates through miRNA family, plant species, UniProt accession number (e.g., [Swiss-Prot:P93015]; SPL3 protein in *Arabidopsis*), and GO term (e.g., rhythmic process). Figure 3B shows an example of the search by UniProt accession number [Swiss-Prot:P93015], where all targeted ESTs annotated with [Swiss-Prot:P93015] will be returned. The "Export Result" button on both the miRNA and Target search result pages allows users to download a search result as a text file containing detailed prediction results of all records.

### Tools for miRNA and miRNA target predictions

**Figure 4:**
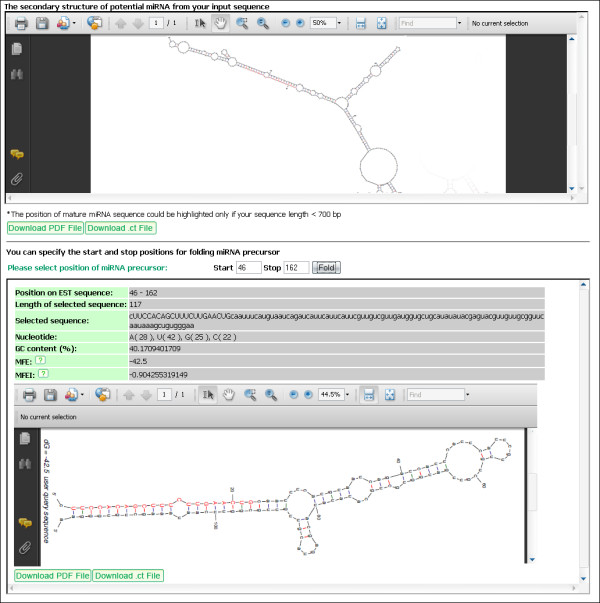
**The secondary structure of an input nucleotide sequence and its substructure of potential pre-miRNA predicted by UNAFold**.

The prediction utility helps to predict whether an input nucleotide sequence (< 3000 bp) is a potential miRNA candidate, and to scan for EST sequences of a plant species potentially targeted by an input mature miRNA sequence (in range of 18–26 nt). For miRNA prediction, users could reduce the number of hit mature miRNAs from miRBase via E-value and the maximum number of alignments setting. It will return all mature miRNA sequences that satisfy setting conditions and found within the input sequence. They are also linked to their potential miRNA homologs in the μPC database. The input sequence with a specific mature miRNA positioned could be further predicted by UNAFold for its secondary structure and substructure of stem-loop with mature miRNA sequence on one arm (Figure 4). To predict miRNA targets, users may reduce the number of possible targets via numbers of allowed mismatches and G:U pairs. Its result includes targeted EST accession numbers from PlantGDB, target site alignment and position, numbers of mismatches and G:U pairs, and its functional annotation.

## Conclusion

The μPC represents the first online resource that facilitates users with the inclusive comparing and predicting plant miRNAs and miRNA targets across plant species and classification. The comparative utility enables users to explore the conservation and evolution of stored miRNAs and user-provided sequences across plant species. Users may explore miRNAs and their targets predicted by large-scale EST analysis via search utility and predict a miRNA or its possible targets for an input sequence via prediction utility. With stored data and incorporated utilities, the μPC conveys a basis for additional research on plant miRNA functions and evolution.

## Availability and requirements

• MicroPC (μPC) is freely available at http://www.biotec.or.th/isl/micropc.

• The comparative miRNA sequence requires Java applet to display output via Jalview.

• The web browser requires a PDF viewer plug-in (e.g., Acrobat Reader) to view and print the PDF files of secondary structures.

## Authors' contributions

WM performed the predictions and developed the database and its web interface. DW provided some annotation results, participated in the design of database, and drafted most of the manuscript. All co-authors read and approved the final manuscript.

## Supplementary Material

Additional file 1 List of plant species and their miRNAs obtained from miRBase (release 12.0). Click here for file

Additional file 2 List of excluded miRBase sequences and their secondary structures. Click here for file

Additional file 3 List of plant species and their EST sequences obtained from PlantGDB (November, 2008) Click here for file

Additional file 4 The miRNA prediction results for the known 936 precursor miRNA sequences from miRBase and the one thousand repeats of 936 random sequences. Click here for file

Additional file 5 The numbers of plant species of each miRNA family obtained from miRBase. Click here for file

Additional file 6 The numbers of correctly predicted precursor miRNAs from miRBase according to the leave-one-out of source mature miRNA sequences. Click here for file
