# Supplementary material for: MicroPC (μPC): A comprehensive resource for predicting and comparing plant microRNAs
Source: BMC Genomics. 2009 Aug 7;10:366. doi: 10.1186/1471-2164-10-366 (PMC2907689; doi:10.1186/1471-2164-10-366)
Supplement: Additional file 1 — List of plant species and their miRNAs obtained from miRBase (release 12.0). [file 1471-2164-10-366-S1.pdf]

List of plant species and their miRNAs obtained from miRBase (release 12.0).

No.	Plant species	Number of miRNA members
1	*Arabidopsis thaliana*	203
2	*Brassica napus*	45
3	*Brassica oleracea*	1
4	*Brassica rapa*	1
5	*Carica papaya*	1
6	*Glycine max*	70
7	*Gossypium herbecium*	1
8	*Gossypium hirsutum*	13
9	*Gossypium rammindii*	2
10	*Medicago truncatula*	30
11	*Oryza sativa*	373
12	*Physcomitrella patens*	263
13	*Pinus taeda*	37
14	*Populus trichocarpa*	237
15	*Saccharum officinarum*	16
16	*Selaginella moellendorffii*	64
17	*Solanum lycopersicum*	30
18	*Sorghum bicolor*	72
19	*Triticum aestivum*	32
20	*Vitis vinifera*	140
21	*Zea mays*	96
Total		1727
