# Supplementary material for: MicroPC (μPC): A comprehensive resource for predicting and comparing plant microRNAs
Source: BMC Genomics. 2009 Aug 7;10:366. doi: 10.1186/1471-2164-10-366 (PMC2907689; doi:10.1186/1471-2164-10-366)
Supplement: Additional file 2 — List of excluded miRBase sequences and their secondary structures. [file 1471-2164-10-366-S2.pdf]

List of excluded miRBase sequences and their secondary structures.

No.	miRBase ID	miRBase AC (mature)	miRBase AC (stem-loop)
1	ghb-MIR169a	MIMAT0005814	MI0005646
2	mtr-MIR169b	MIMAT0001644	MI0001742
3	mtr-MIR395h	MIMAT0003861	MI0005075
4	mtr-MIR395i	MIMAT0003862	MI0005076
5	mtr-MIR395j	MIMAT0003863	MI0005077
6	mtr-MIR395p	MIMAT0003869	MI0005083
7	mtr-MIR399a	MIMAT0001651	MI0001749
8	mtr-MIR399e	MIMAT0001652	MI0001750
9	osa-MIR156a	MIMAT0000618	MI0000653
10	osa-MIR156c	MIMAT0000620	MI0000655
11	osa-MIR156d	MIMAT0000621	MI0000656
12	osa-MIR156e	MIMAT0000622	MI0000657
13	osa-MIR156f	MIMAT0000623	MI0000658
14	osa-MIR156g	MIMAT0000624	MI0000659
15	osa-MIR156h	MIMAT0000625	MI0000660
16	osa-MIR156i	MIMAT0000626	MI0000661
17	osa-MIR156j	MIMAT0000627	MI0000662
18	osa-MIR156l	MIMAT0001021	MI0001091
19	osa-MIR159b	MIMAT0001023	MI0001093
20	osa-MIR166g	MIMAT0001072	MI0001142
21	osa-MIR166i	MIMAT0001074	MI0001144
22	osa-MIR166j	MIMAT0001036	MI0001106
23	osa-MIR166k	MIMAT0001037	MI0001107
24	osa-MIR166m	MIMAT0001087	MI0001157
25	osa-MIR168b	MIMAT0001046	MI0001116
26	osa-MIR169e	MIMAT0001050	MI0001120
27	osa-MIR169f	MIMAT0001051	MI0001121
28	osa-MIR169g	MIMAT0001052	MI0001122
29	osa-MIR169h	MIMAT0001053	MI0001123
30	osa-MIR169i	MIMAT0001054	MI0001124
31	osa-MIR169j	MIMAT0001055	MI0001125
32	osa-MIR169k	MIMAT0001056	MI0001126
33	osa-MIR169l	MIMAT0001057	MI0001127
34	osa-MIR169m	MIMAT0001058	MI0001128
35	osa-MIR169q	MIMAT0001062	MI0001132
36	osa-MIR172a	MIMAT0001069	MI0001139
37	osa-MIR395a	MIMAT0000973	MI0001042
38	osa-MIR395m	MIMAT0003870	MI0005084
39	osa-MIR395t	MIMAT0000969	MI0001038
40	osa-MIR395v	MIMAT0003876	MI0005090
41	osa-MIR395w	MIMAT0003877	MI0005091
42	osa-MIR397b	MIMAT0000981	MI0001050
43	osa-MIR399e	MIMAT0000988	MI0001057
44	osa-MIR399f	MIMAT0000989	MI0001058
45	osa-MIR399j	MIMAT0000993	MI0001062
46	osa-MIR399k	MIMAT0000994	MI0001063
47	osa-MIR408	MIMAT0001079	MI0001149
48	osa-MIR413	MIMAT0001329	MI0001433
49	osa-MIR414	MIMAT0001330	MI0001434
50	osa-MIR415	MIMAT0001331	MI0001435
51	osa-MIR416	MIMAT0001332	MI0001436
52	osa-MIR417	MIMAT0001333	MI0001437
53	osa-MIR418	MIMAT0001334	MI0001438
54	osa-MIR419	MIMAT0001335	MI0001439
55	osa-MIR426	MIMAT0001338	MI0001442
56	ppt-MIR167	MIMAT0004353	MI0005661
57	pta-MIR159c	MIMAT0004992	MI0005780
58	pta-MIR166c	MIMAT0004995	MI0005784
59	pta-MIR171	MIMAT0004996	MI0005785
60	pta-MIR408	MIMAT0005001	MI0005790

List of excluded miRBase sequences and their secondary structures (cont.).

No.	miRBase ID	miRBase AC (mature)	miRBase AC (stem-loop)
61	pta-MIR482a	MIMAT0005002	MI0005791
62	pta-MIR482b	MIMAT0005003	MI0005792
63	pta-MIR482c	MIMAT0006014	MI0007052
64	pta-MIR482d	MIMAT0006015	MI0007053
65	pta-MIR783	MIMAT0005004	MI0005793
66	ptc-MIR156c	MIMAT0001892	MI0002186
67	ptc-MIR156e	MIMAT0001894	MI0002188
68	ptc-MIR159d	MIMAT0001904	MI0002198
69	ptc-MIR160g	MIMAT0001913	MI0002207
70	ptc-MIR166i	MIMAT0001932	MI0002226
71	ptc-MIR166n	MIMAT0001937	MI0002231
72	ptc-MIR166o	MIMAT0001938	MI0002232
73	ptc-MIR166p	MIMAT0001939	MI0002233
74	ptc-MIR166q	MIMAT0001940	MI0002234
75	ptc-MIR169ac	MIMAT0001954	MI0002248
76	ptc-MIR169ad	MIMAT0001955	MI0002249
77	ptc-MIR169d	MIMAT0001960	MI0002254
78	ptc-MIR169e	MIMAT0001961	MI0002255
79	ptc-MIR169h	MIMAT0001964	MI0002258
80	ptc-MIR169i	MIMAT0001965	MI0002259
81	ptc-MIR169j	MIMAT0001966	MI0002260
82	ptc-MIR169k	MIMAT0001967	MI0002261
83	ptc-MIR169l	MIMAT0001968	MI0002262
84	ptc-MIR169m	MIMAT0001969	MI0002263
85	ptc-MIR169q	MIMAT0001973	MI0002267
86	ptc-MIR169u	MIMAT0001977	MI0002271
87	ptc-MIR169v	MIMAT0001978	MI0002272
88	ptc-MIR169w	MIMAT0001979	MI0002273
89	ptc-MIR169x	MIMAT0001980	MI0002274
90	ptc-MIR171j	MIMAT0001992	MI0002286
91	ptc-MIR172d	MIMAT0001996	MI0002290
92	ptc-MIR172f	MIMAT0001998	MI0002292
93	ptc-MIR172i	MIMAT0002001	MI0002295
94	ptc-MIR397c	MIMAT0002040	MI0002334
95	ptc-MIR399a	MIMAT0002044	MI0002338
96	ptc-MIR399b	MIMAT0002045	MI0002339
97	ptc-MIR399c	MIMAT0002046	MI0002340
98	ptc-MIR399g	MIMAT0002050	MI0002344
99	ptc-MIR399j	MIMAT0002053	MI0002347
100	ptc-MIR399l	MIMAT0002055	MI0002349
101	ptc-MIR474b	MIMAT0002065	MI0002359
102	ptc-MIR474c	MIMAT0002066	MI0002360
103	ptc-MIR478e	MIMAT0002080	MI0002374
104	ptc-MIR478f	MIMAT0002081	MI0002375
105	ptc-MIR478h	MIMAT0002082	MI0002376
106	ptc-MIR478i	MIMAT0002083	MI0002377
107	ptc-MIR478j	MIMAT0002084	MI0002378
108	ptc-MIR478k	MIMAT0002085	MI0002379
109	ptc-MIR478l	MIMAT0002086	MI0002380
110	ptc-MIR478m	MIMAT0002087	MI0002381
111	ptc-MIR478n	MIMAT0002088	MI0002382
112	ptc-MIR478o	MIMAT0002089	MI0002383
113	ptc-MIR478p	MIMAT0002090	MI0002384
114	ptc-MIR478q	MIMAT0002091	MI0002385
115	ptc-MIR478r	MIMAT0002092	MI0002386
116	ptc-MIR478s	MIMAT0002093	MI0002387
117	ptc-MIR478u	MIMAT0002094	MI0002388
118	ptc-MIR480b	MIMAT0002097	MI0002391
119	sbi-MIR156a	MIMAT0001398	MI0001504

List of excluded miRBase sequences and their secondary structures (cont.).

No.	miRBase ID	miRBase AC (mature)	miRBase AC (stem-loop)
120	sbi-MIR156c	MIMAT0001399	MI0001505
121	sbi-MIR156e	MIMAT0001758	MI0001856
122	sbi-MIR159b	MIMAT0001753	MI0001851
123	sbi-MIR160b	MIMAT0001404	MI0001510
124	sbi-MIR160e	MIMAT0001405	MI0001511
125	sbi-MIR164	MIMAT0001406	MI0001512
126	sbi-MIR166g	MIMAT0001755	MI0001853
127	sbi-MIR169c	MIMAT0001453	MI0001557
128	sbi-MIR169d	MIMAT0001454	MI0001558
129	sbi-MIR169e	MIMAT0001455	MI0001559
130	sbi-MIR169f	MIMAT0001456	MI0001560
131	sbi-MIR169g	MIMAT0001457	MI0001561
132	sbi-MIR169h	MIMAT0001458	MI0001562
133	sbi-MIR394b	MIMAT0001428	MI0001532
134	sbi-MIR395a	MIMAT0001430	MI0001534
135	sbi-MIR399e	MIMAT0001440	MI0001544
136	sbi-MIR399f	MIMAT0001441	MI0001545
137	sbi-MIR399g	MIMAT0001443	MI0001547
138	sly-MIR162	MIMAT0009142	MI0009975
139	sof-MIR156	MIMAT0001656	MI0001754
140	vvi-MIR156b	MIMAT0005641	MI0006486
141	vvi-MIR156d	MIMAT0005643	MI0006488
142	vvi-MIR156e	MIMAT0005644	MI0006489
143	vvi-MIR156h	MIMAT0006544	MI0007939
144	vvi-MIR160a	MIMAT0005651	MI0006496
145	vvi-MIR164a	MIMAT0005658	MI0006503
146	vvi-MIR164b	MIMAT0005659	MI0006504
147	vvi-MIR164c	MIMAT0005660	MI0006505
148	vvi-MIR166a	MIMAT0005662	MI0006507
149	vvi-MIR166c	MIMAT0005664	MI0006509
150	vvi-MIR166d	MIMAT0005665	MI0006510
151	vvi-MIR166e	MIMAT0005666	MI0006511
152	vvi-MIR166h	MIMAT0005669	MI0006514
153	vvi-MIR169e	MIMAT0005680	MI0006525
154	vvi-MIR169f	MIMAT0005681	MI0006526
155	vvi-MIR169h	MIMAT0006546	MI0007941
156	vvi-MIR169n	MIMAT0006549	MI0007944
157	vvi-MIR169x	MIMAT0006554	MI0007949
158	vvi-MIR169y	MIMAT0005677	MI0006522
159	vvi-MIR171g	MIMAT0006555	MI0007950
160	vvi-MIR172a	MIMAT0005699	MI0006544
161	vvi-MIR319c	MIMAT0005704	MI0006549
162	vvi-MIR399f	MIMAT0006567	MI0007962
163	vvi-MIR399g	MIMAT0005731	MI0006576
164	vvi-MIR403c	MIMAT0006571	MI0007966
165	vvi-MIR477	MIMAT0006575	MI0007970
166	vvi-MIR828b	MIMAT0006578	MI0007973
167	vvi-MIR845a	MIMAT0006579	MI0007974
168	vvi-MIR845b	MIMAT0006580	MI0007975
169	vvi-MIR845c	MIMAT0006581	MI0007976
170	vvi-MIR845d	MIMAT0006582	MI0007977
171	vvi-MIR845e	MIMAT0006583	MI0007978
172	zma-MIR156a	MIMAT0001357	MI0001462
173	zma-MIR156c	MIMAT0001355	MI0001460
174	zma-MIR156d	MIMAT0001351	MI0001456
175	zma-MIR156e	MIMAT0001356	MI0001461
176	zma-MIR156f	MIMAT0001352	MI0001457
177	zma-MIR156g	MIMAT0001353	MI0001458
178	zma-MIR156h	MIMAT0001358	MI0001463

List of excluded miRBase sequences and their secondary structures (cont.).

No.	miRBase ID	miRBase AC (mature)	miRBase AC (stem-loop)
179	zma-MIR156i	MIMAT0001359	MI0001464
180	zma-MIR156k	MIMAT0001749	MI0001847
181	zma-MIR166k	MIMAT0001719	MI0001817
182	zma-MIR169c	MIMAT0001728	MI0001826
183	zma-MIR169d	MIMAT0001735	MI0001833
184	zma-MIR169e	MIMAT0001736	MI0001834
185	zma-MIR169g	MIMAT0001730	MI0001828
186	zma-MIR169h	MIMAT0001731	MI0001829
187	zma-MIR169i	MIMAT0001732	MI0001830
188	zma-MIR169j	MIMAT0001734	MI0001832
189	zma-MIR169k	MIMAT0001733	MI0001831
190	zma-MIR172e	MIMAT0001742	MI0001840
191	zma-MIR395a	MIMAT0001701	MI0001799
192	zma-MIR408	MIMAT0001748	MI0001846

Notes for secondary structure figures:

1. The mature miRNA sequence is represented in red capital letters.
2. The name of each figure includes miRBase ID and AC separated by "_".

Output of sir_graph (©)
by D. Stewart and M. Zuker

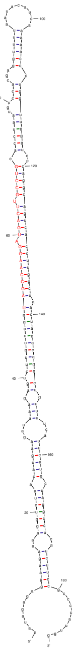

ghb-MIR169a_MI0005646_MIMAT0005814

Output of sir_graph (©)
by D. Stewart and M. Zuker

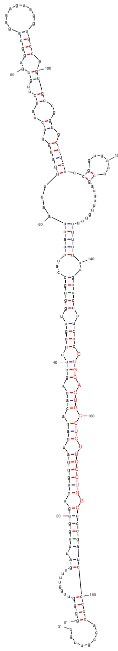

zma-MIR408_MI0001846_MIMAT0001748

Output of sir_graph (©)
by D. Stewart and M. Zuker

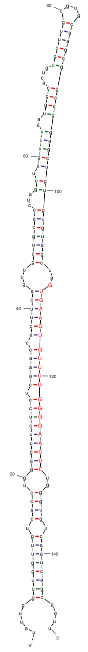

zma-MIR395a_MI0001799_MIMAT0001701

Output of sir_graph (©)
by D. Stewart and M. Zuker

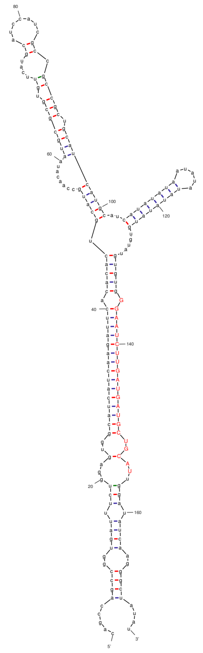

zma-MIR172e_MI0001840_MIMAT0001742

Output of sir_graph (©)
by D. Stewart and M. Zuker

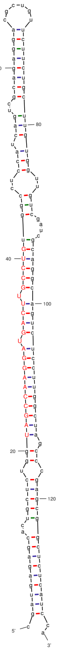

zma-MIR169k_MI0001831_MIMAT0001733

Output of sir_graph (©)
by D. Stewart and M. Zuker

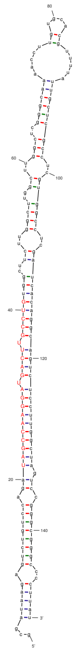

zma-MIR169j_MI0001832_MIMAT0001734

Output of sir_graph (©)
by D. Stewart and M. Zuker

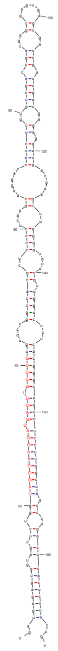

zma-MIR169i_MI0001830_MIMAT0001732

Output of sir_graph (©)
by D. Stewart and M. Zuker

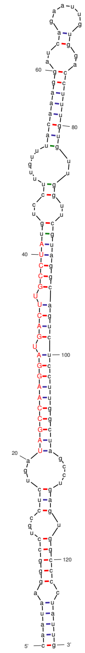

zma-MIR169h_MI0001829_MIMAT0001731

Output of sir_graph (©)
by D. Stewart and M. Zuker

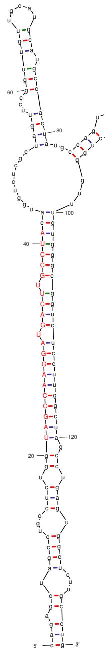

zma-MIR169g_MI0001828_MIMAT0001730

Output of sir_graph (fi)
by D. Stewart and M. Zuker

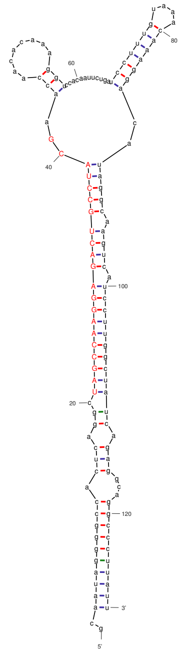

zma-MIR169e_MI0001834_MIMAT0001736

Output of sir_graph (fi)
by D. Stewart and M. Zuker

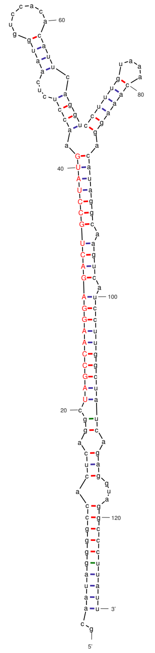

zma-MIR169d_MI0001833_MIMAT0001735

Output of sir_graph (fi)
by D. Stewart and M. Zuker

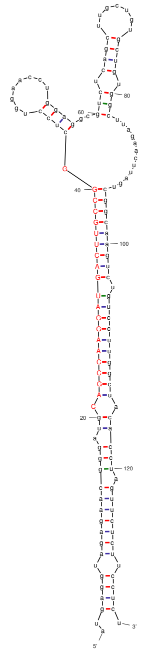

zma-MIR169c_MI0001826_MIMAT0001728

Output of sir_graph (fi)
by D. Stewart and M. Zuker

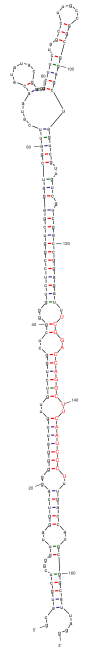

zma-MIR166k_MI0001817_MIMAT0001719

Output of sir_graph (fi)
by D. Stewart and M. Zuker

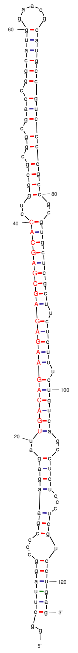

zma-MIR156k_MI0001847_MIMAT0001749

Output of sir_graph (fi)
by D. Stewart and M. Zuker

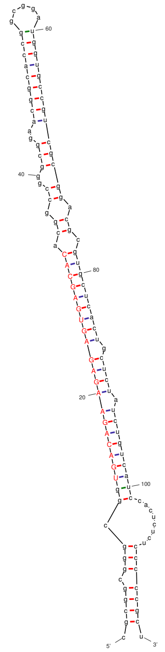

zma-MIR156i_MI0001464_MIMAT0001359

Output of sir_graph (fi)
by D. Stewart and M. Zuker

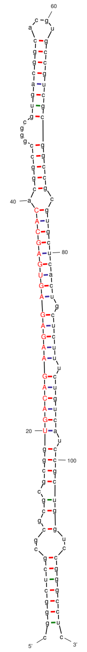

zma-MIR156h_MI0001463_MIMAT0001358

Output of sir_graph (fi)
by D. Stewart and M. Zuker

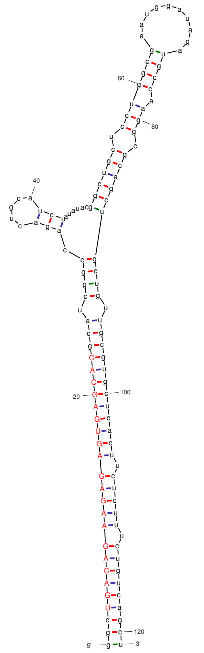

zma-MIR156g_MI0001458_MIMAT0001353

Output of sir_graph (fi)
by D. Stewart and M. Zuker

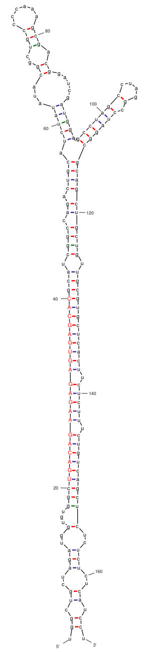

zma-MIR156f_MI0001457_MIMAT0001352

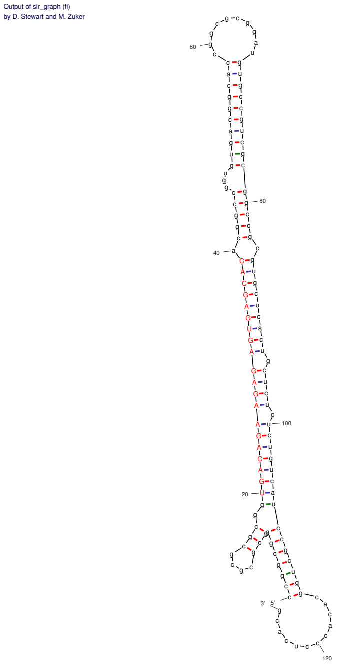

zma-MIR156e_MI0001461_MIMAT0001356

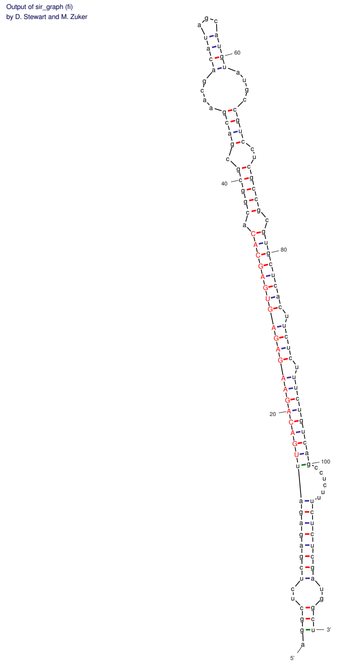

zma-MIR156d_MI0001456_MIMAT0001351

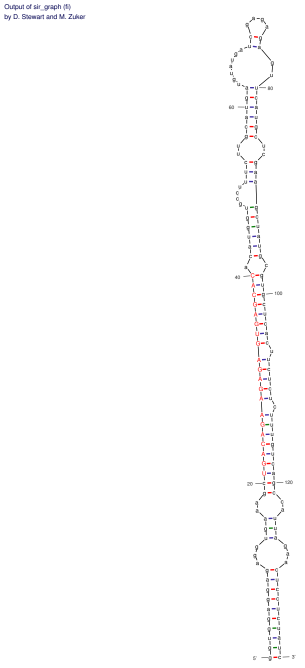

zma-MIR156c_MI0001460_MIMAT0001355

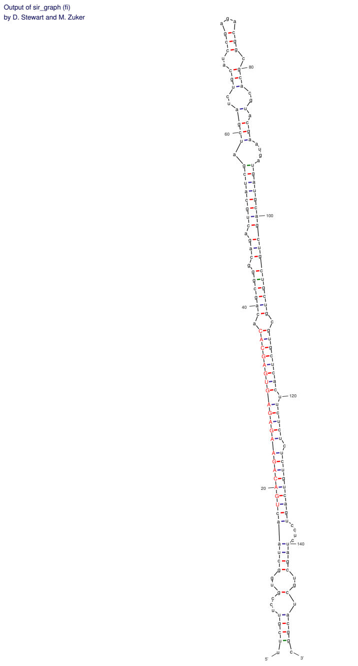

zma-MIR156a_MI0001462_MIMAT0001357

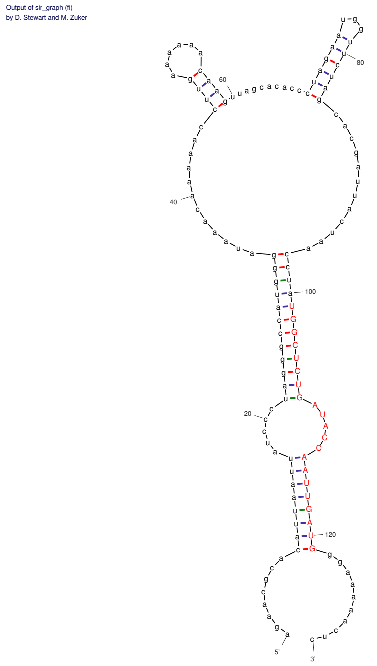

vvi-MIR845e_MI0007978_MIMAT0006583

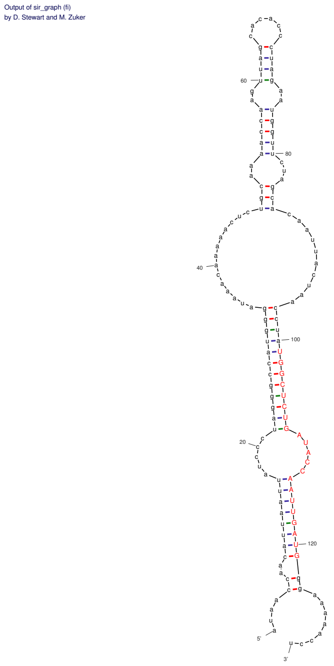

vvi-MIR845d_MI0007977_MIMAT0006582

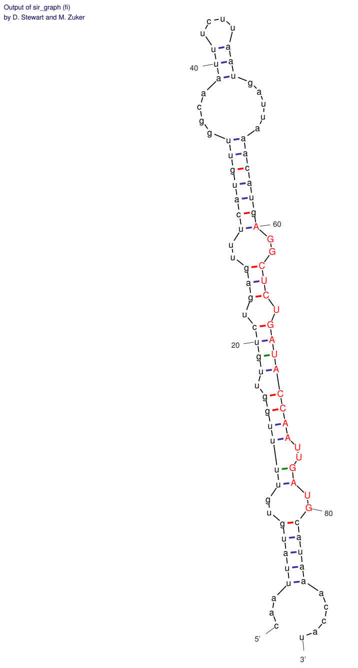

vvi-MIR845c_MI0007976_MIMAT0006581

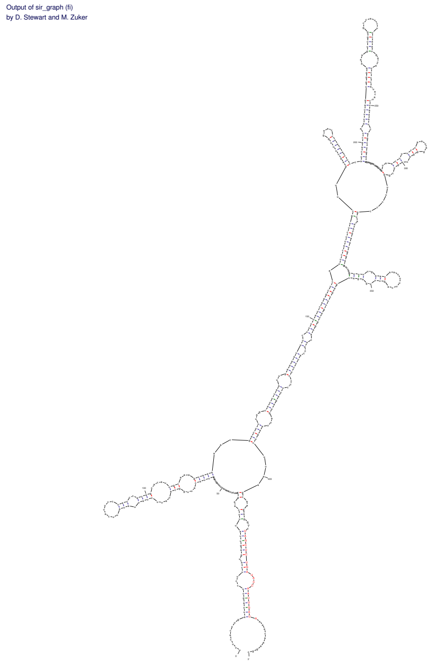

vvi-MIR845b_MI0007975_MIMAT0006580

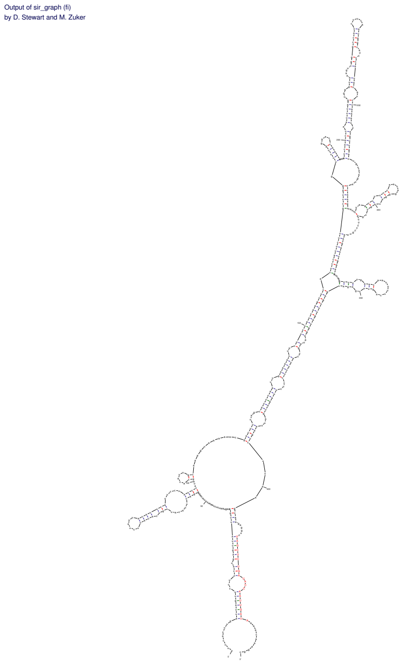

vvi-MIR845a_MI0007974_MIMAT0006579

Output of sir_graph (©)
by D. Stewart and M. Zuker

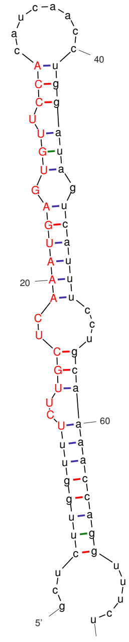

vvi-MIR828b_MI0007973_MIMAT0006578

Output of sir_graph (©)
by D. Stewart and M. Zuker

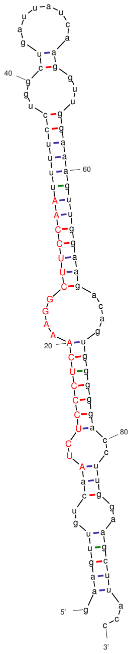

vvi-MIR477_MI0007970_MIMAT0006575

Output of sir_graph (©)
by D. Stewart and M. Zuker

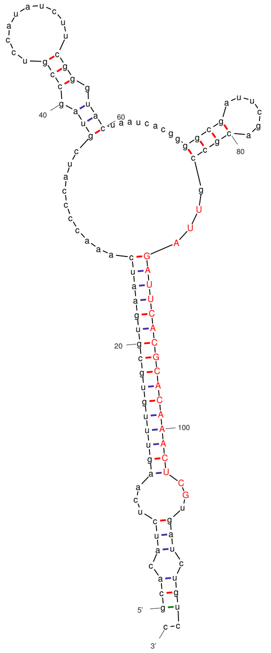

vvi-MIR403c_MI0007966_MIMAT0006571

Output of sir_graph (©)
by D. Stewart and M. Zuker

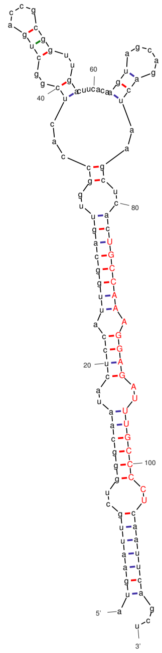

vvi-MIR399g_MI0006576_MIMAT0005731

Output of sir_graph (©)
by D. Stewart and M. Zuker

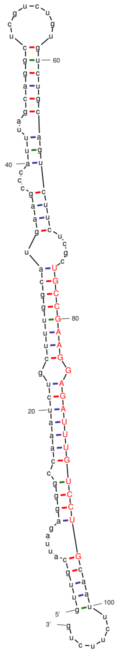

vvi-MIR399f_MI0007962_MIMAT0006567

Output of sir_graph (©)
by D. Stewart and M. Zuker

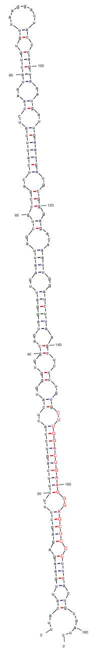

vvi-MIR319c_MI0006549_MIMAT0005704

Output of sir_graph (©)
by D. Stewart and M. Zuker

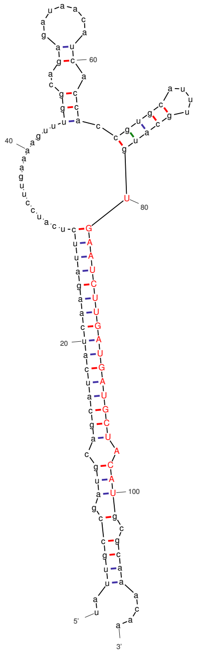

vvi-MIR172a_MI0006544_MIMAT0005699

Output of sir_graph (©)
by D. Stewart and M. Zuker

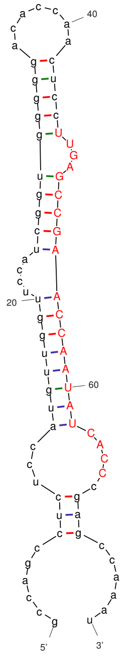

vvi-MIR171g_MI0007950_MIMAT0006555

Output of sir_graph (©)
by D. Stewart and M. Zuker

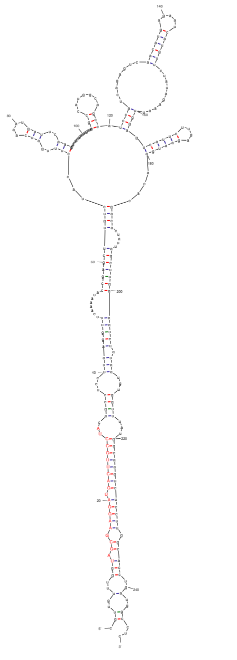

vvi-MIR169y_MI0006522_MIMAT0005677

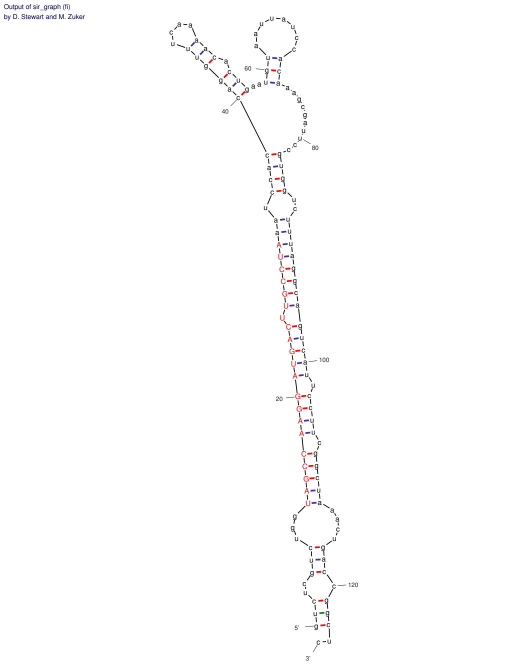

vvi-MIR169x_MI0007949_MIMAT0006554

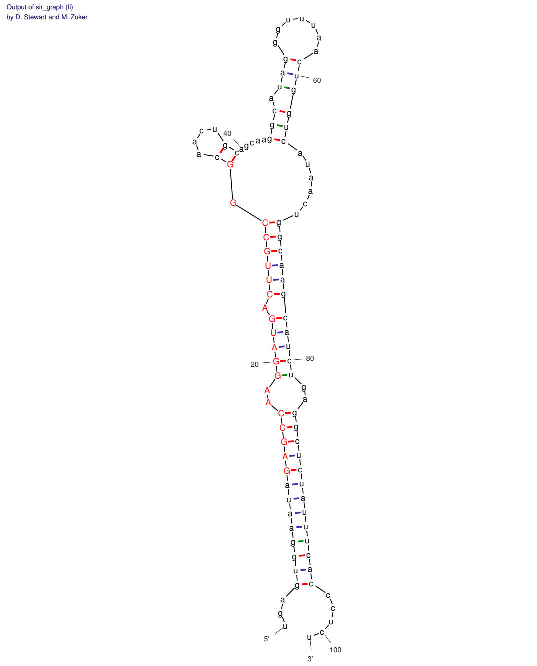

vvi-MIR169n_MI0007944_MIMAT0006549

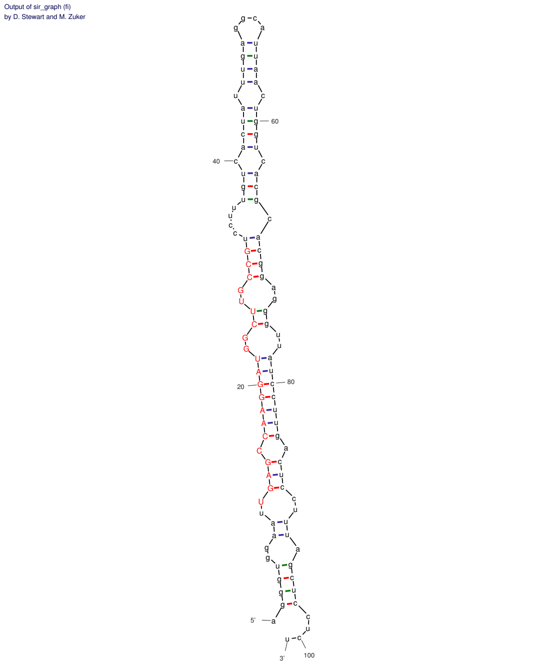

vvi-MIR169h_MI0007941_MIMAT0006546

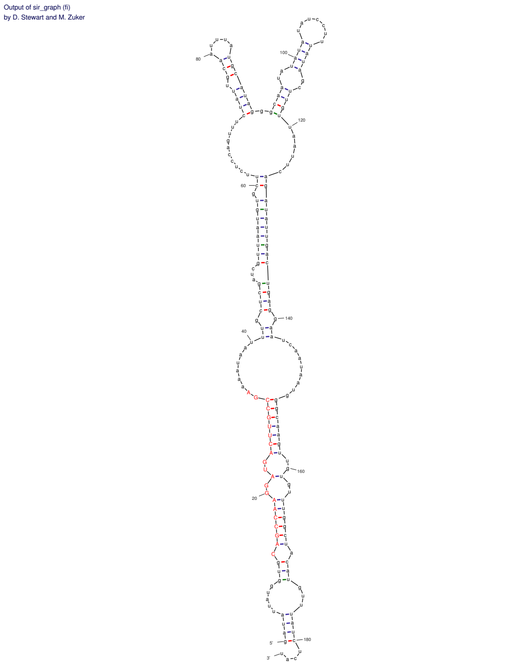

vvi-MIR169f_MI0006526_MIMAT0005681

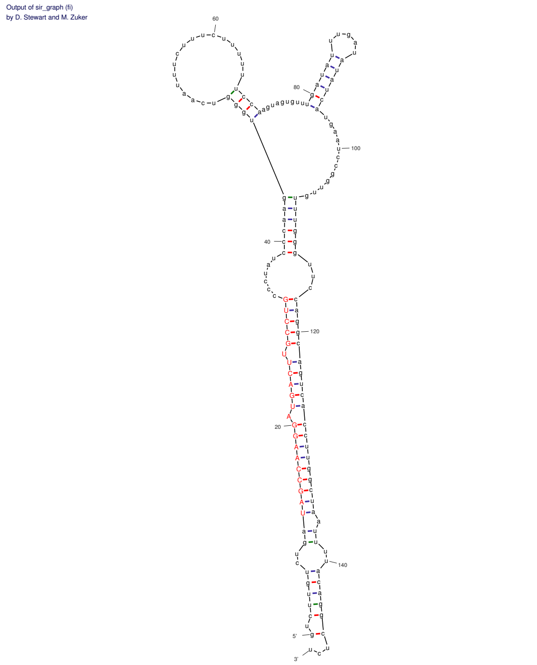

vvi-MIR169e_MI0006525_MIMAT0005680

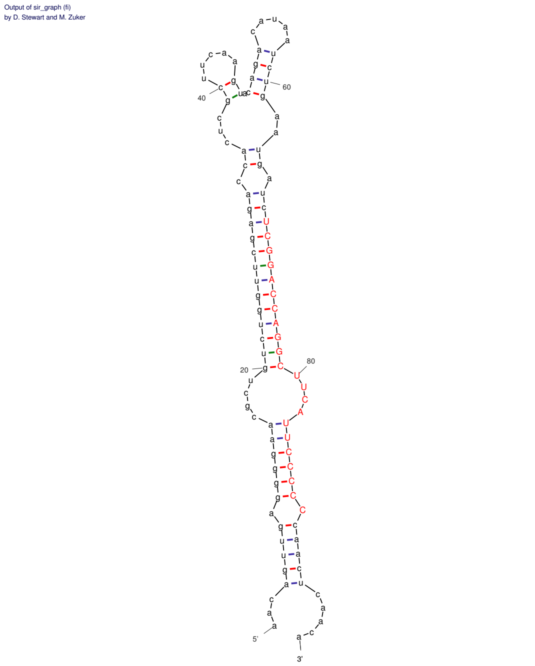

vvi-MIR166h_MI0006514_MIMAT0005669

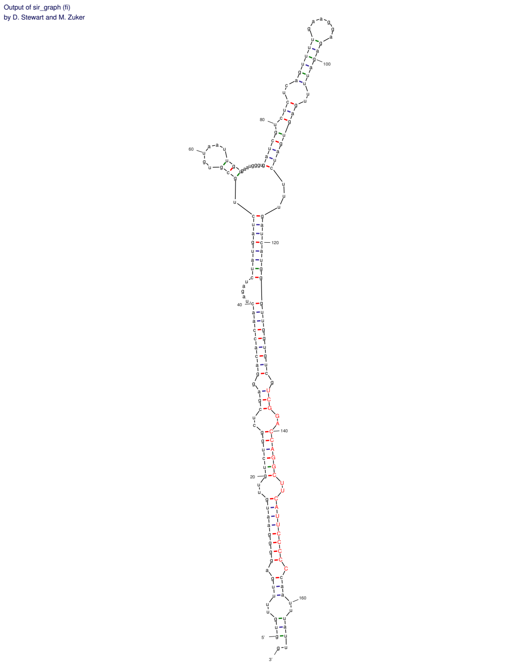

vvi-MIR166e_MI0006511_MIMAT0005666

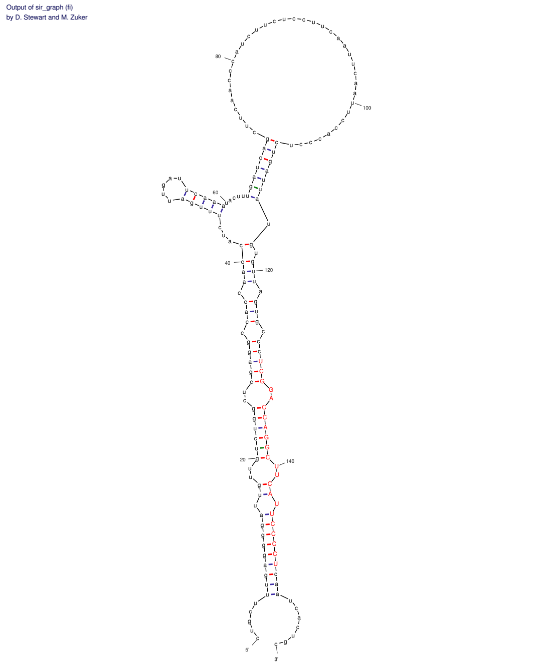

vvi-MIR166d_MI0006510_MIMAT0005665

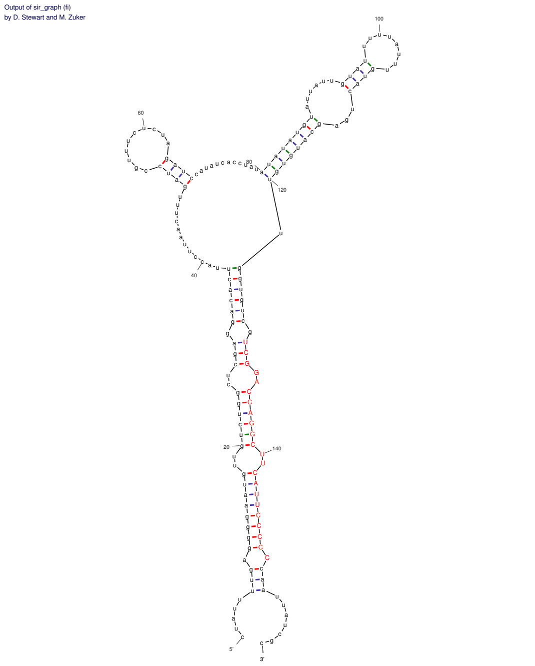

vvi-MIR166c_MI0006509_MIMAT0005664

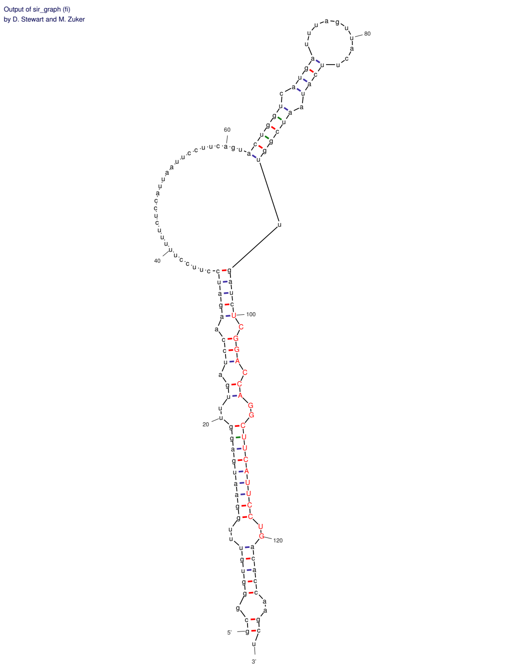

vvi-MIR166a_MI0006507_MIMAT0005662

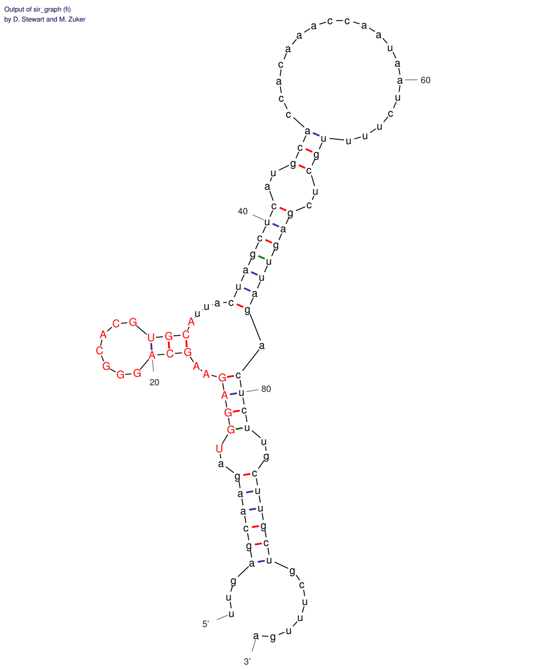

vvi-MIR164c_MI0006505_MIMAT0005660

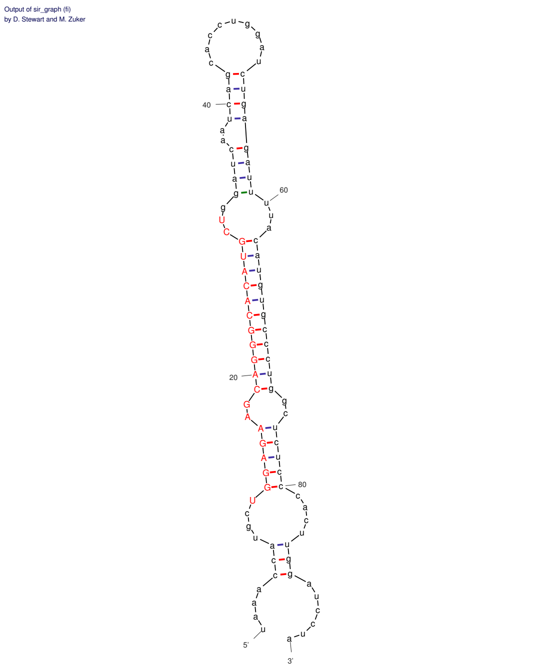

vvi-MIR164b_MI0006504_MIMAT0005659

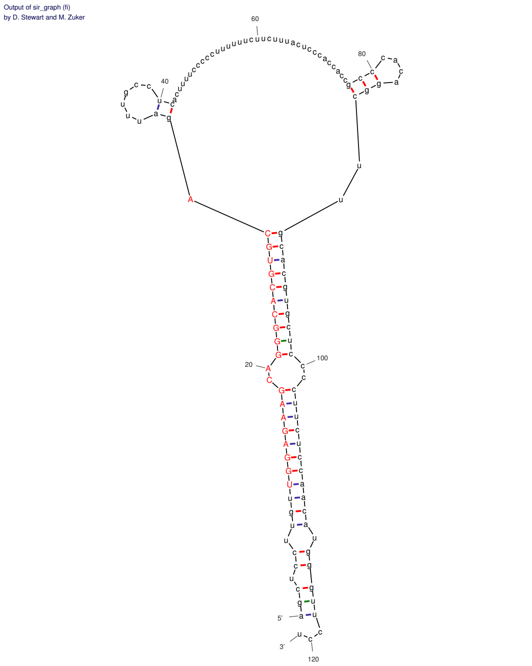

vvi-MIR164a_MI0006503_MIMAT0005658

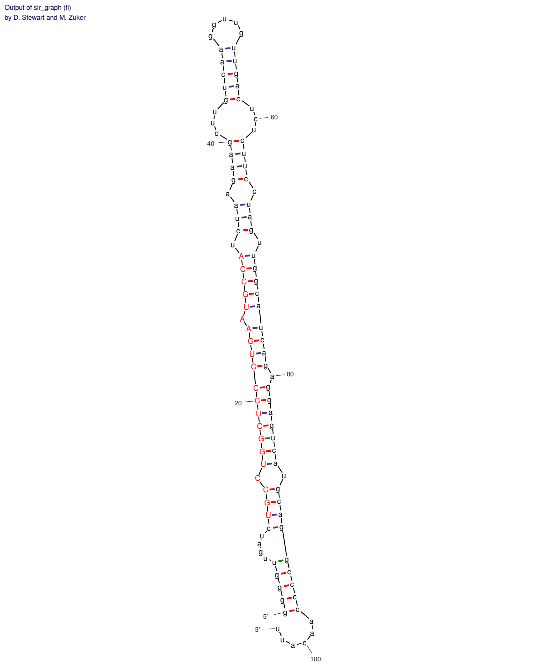

vvi-MIR160a_MI0006496_MIMAT0005651

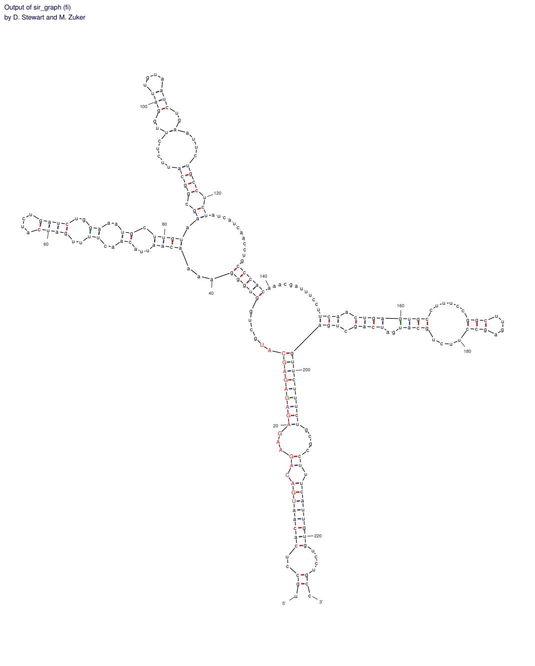

vvi-MIR156h_MI0007939_MIMAT0006544

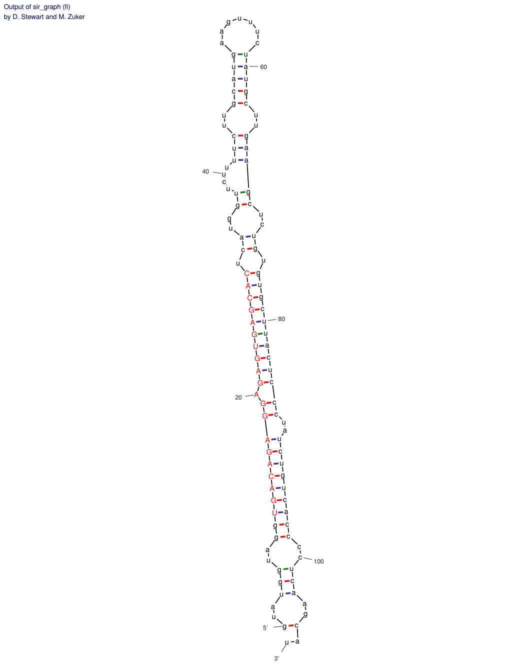

vvi-MIR156e_MI0006489_MIMAT0005644

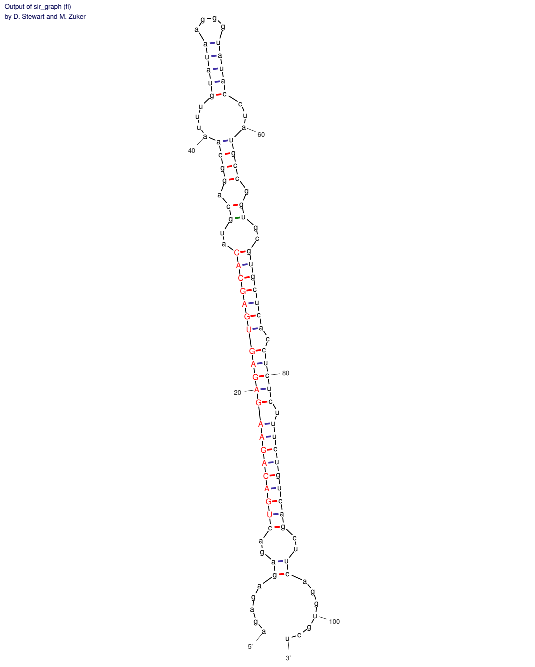

vvi-MIR156d_MI0006488_MIMAT0005643

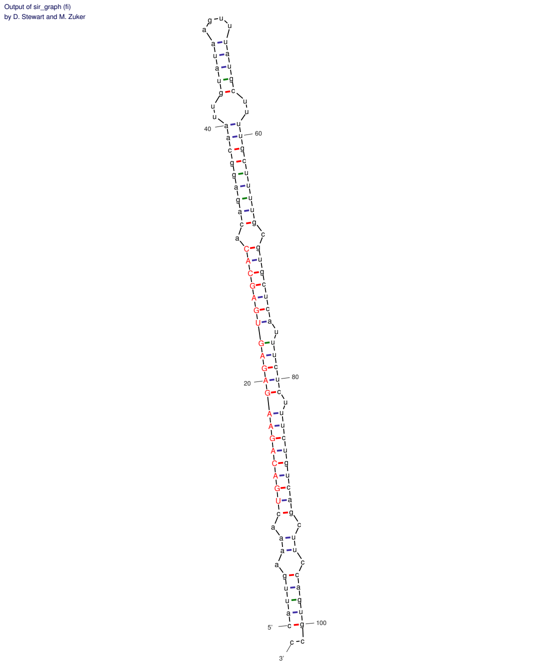

vvi-MIR156b_MI0006486_MIMAT0005641

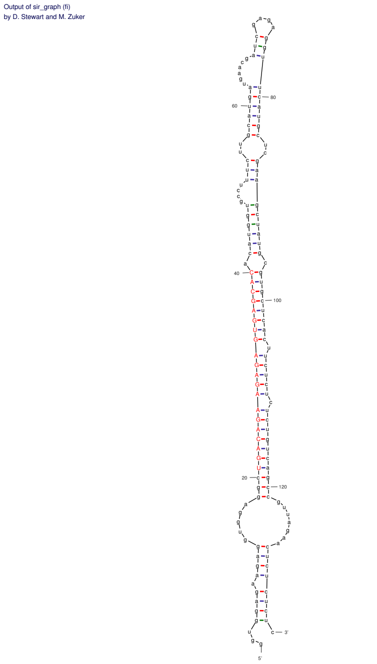

sof-MIR156_MI0001754_MIMAT0001656

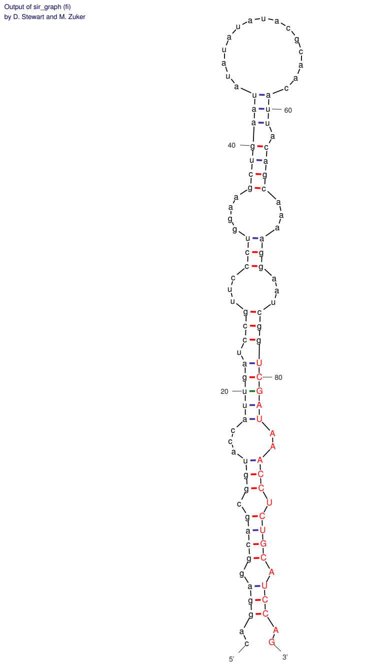

sly-MIR162_MI0009975_MIMAT0009142

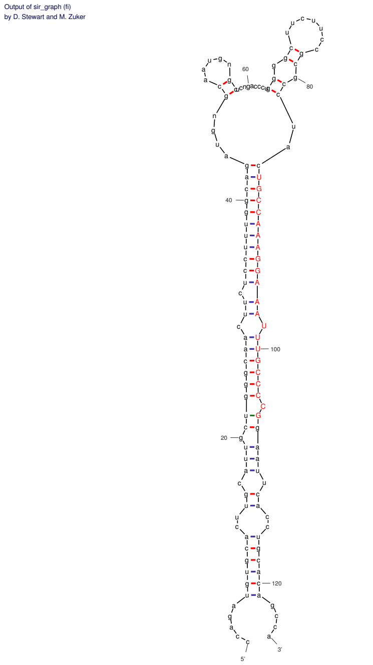

sbi-MIR399g_MI0001547_MIMAT0001443

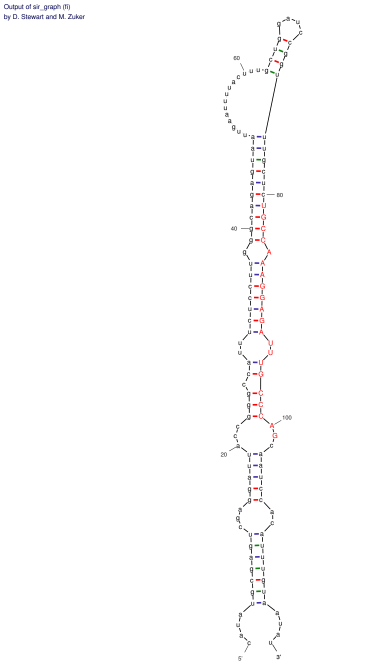

sbi-MIR399f_MI0001545_MIMAT0001441

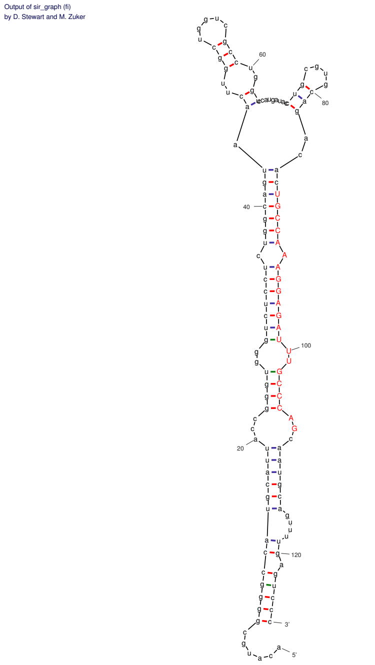

sbi-MIR399e_MI0001544_MIMAT0001440

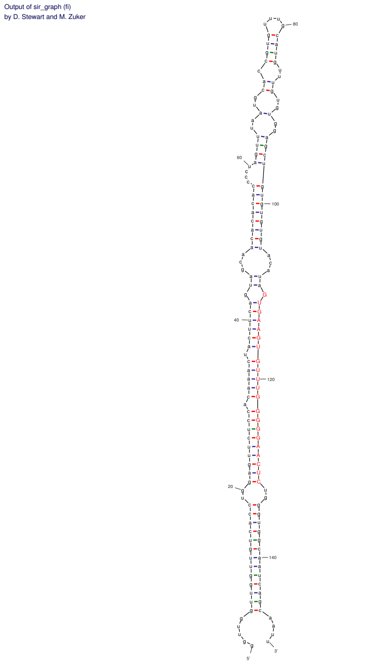

sbi-MIR395a_MI0001534_MIMAT0001430

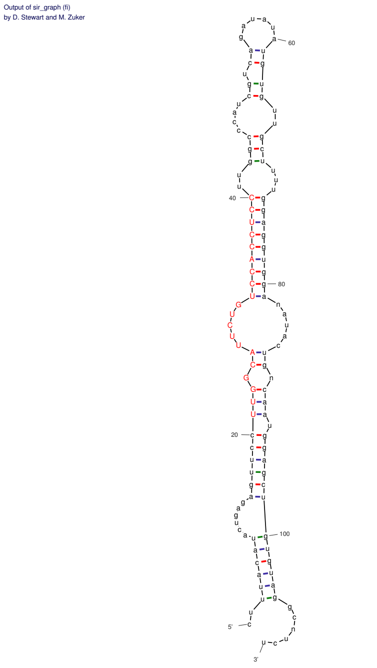

sbi-MIR394b_MI0001532_MIMAT0001428

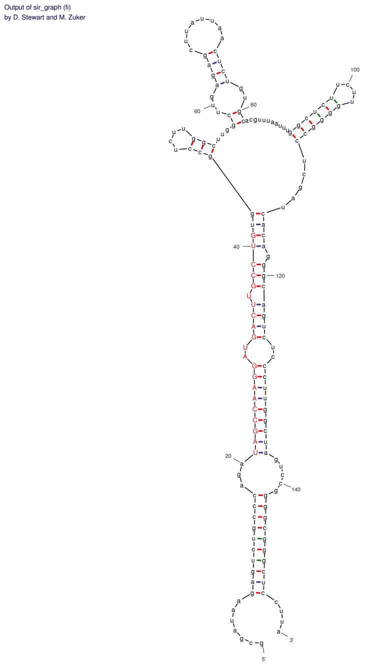

sbi-MIR169h_MI0001562_MIMAT0001458

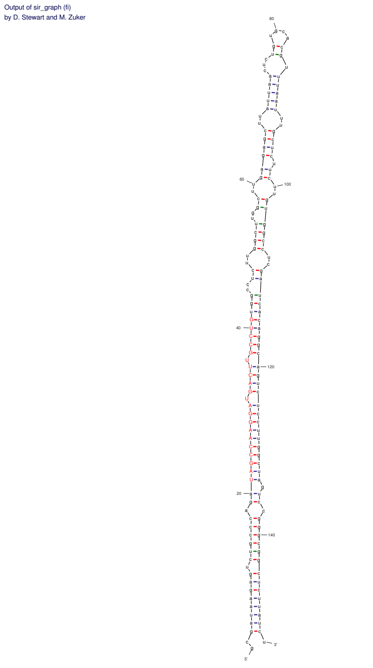

sbi-MIR169g_MI0001561_MIMAT0001457

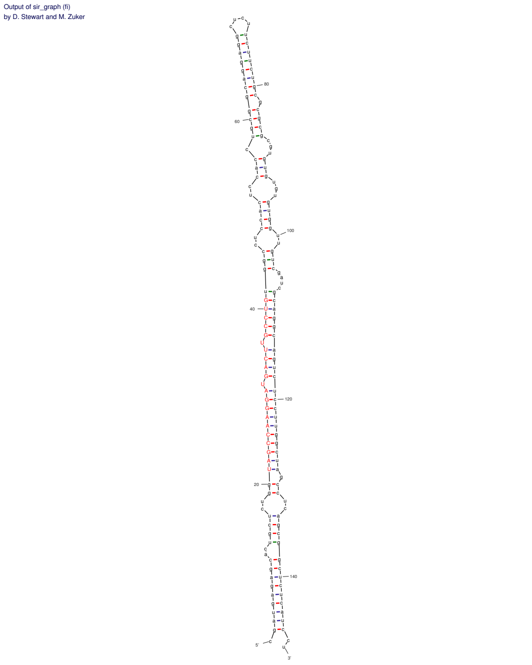

sbi-MIR169f_MI0001560_MIMAT0001456

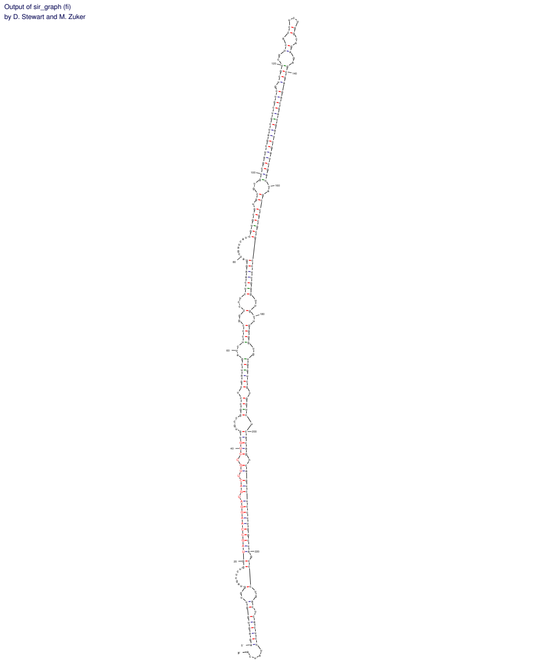

sbi-MIR169e_MI0001559_MIMAT0001455

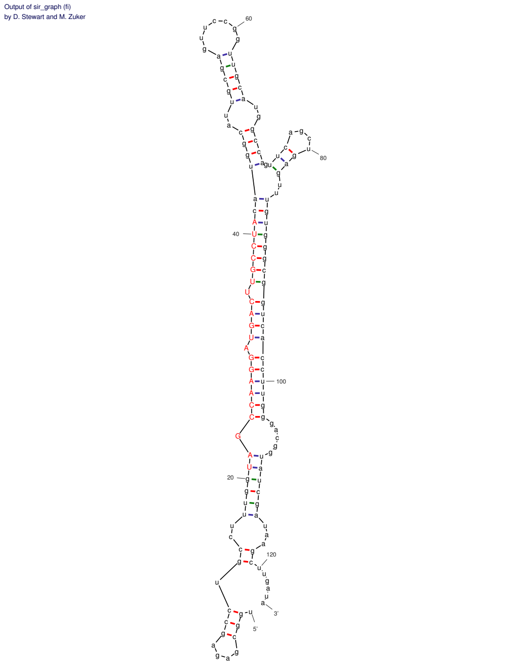

sbi-MIR169d_MI0001558_MIMAT0001454

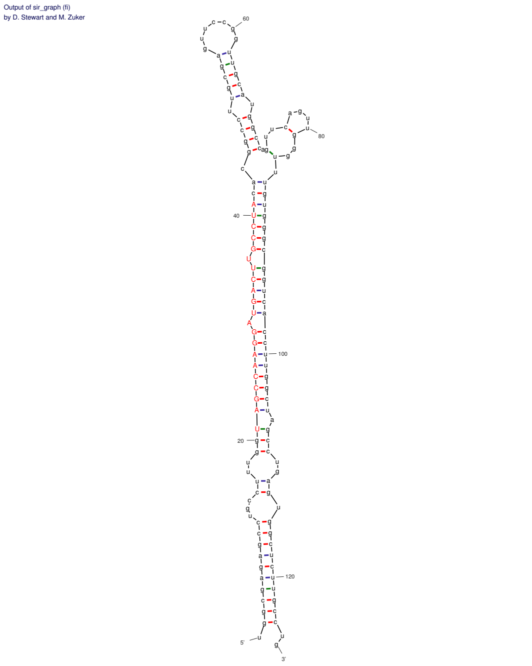

sbi-MIR169c_MI0001557_MIMAT0001453

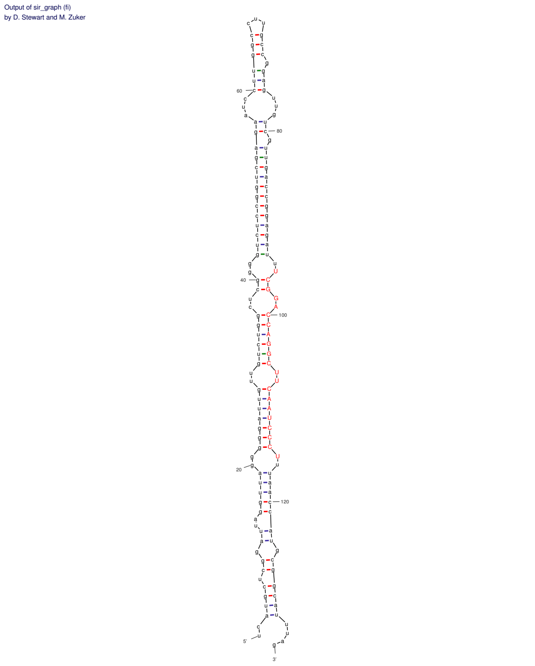

sbi-MIR166g_MI0001853_MIMAT0001755

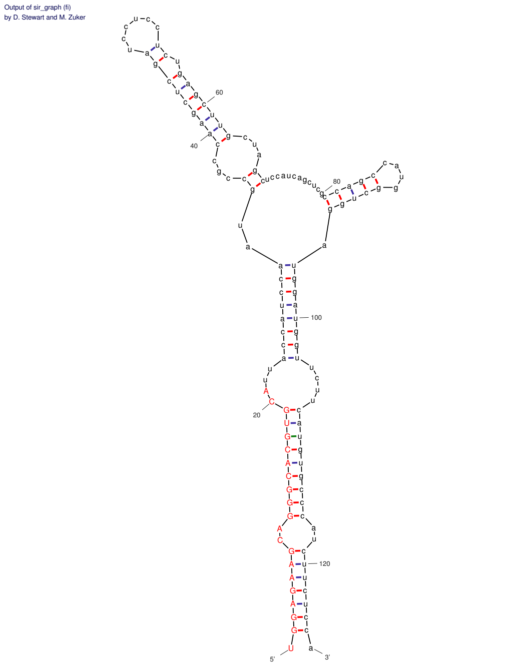

sbi-MIR164_MI0001512_MIMAT0001406

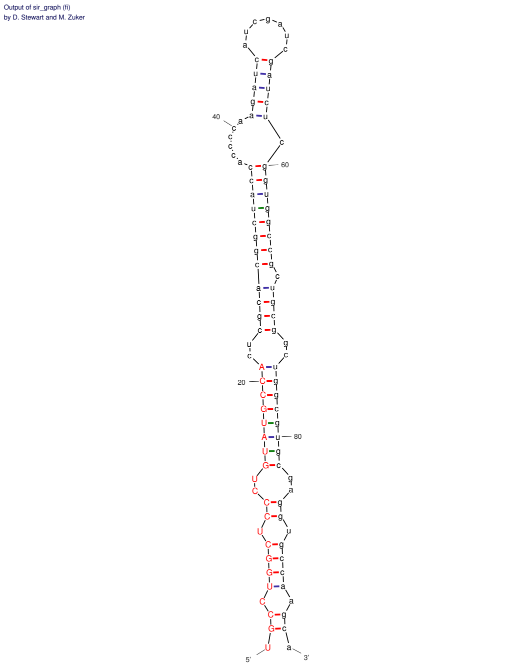

sbi-MIR160e_MI0001511_MIMAT0001405

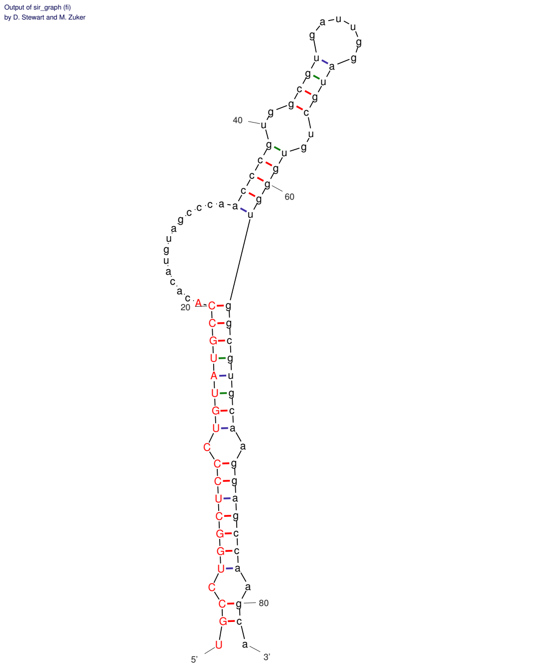

sbi-MIR160b_MI0001510_MIMAT0001404

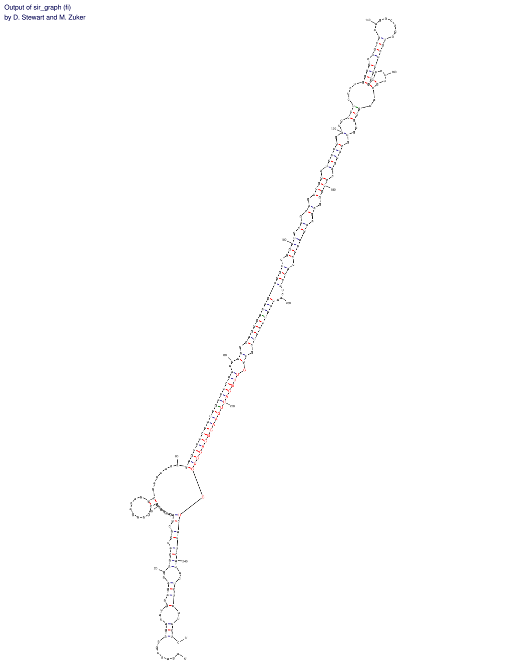

sbi-MIR159b_MI0001851_MIMAT0001753

Output of sir_graph (fl)
by D. Stewart and M. Zuker

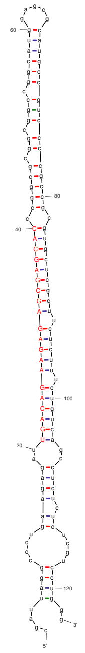

sbi-MIR156e_MI0001856_MIMAT0001758

Output of sir_graph (fl)
by D. Stewart and M. Zuker

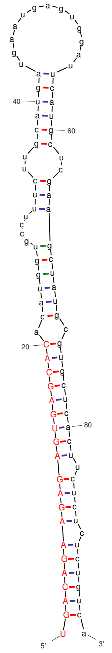

sbi-MIR156c_MI0001505_MIMAT0001399

Output of sir_graph (fl)
by D. Stewart and M. Zuker

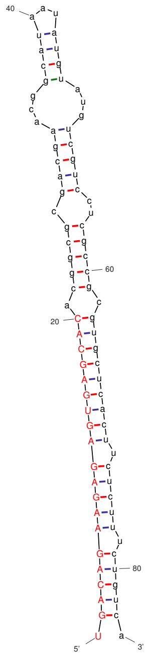

sbi-MIR156a_MI0001504_MIMAT0001398

Output of sir_graph (fl)
by D. Stewart and M. Zuker

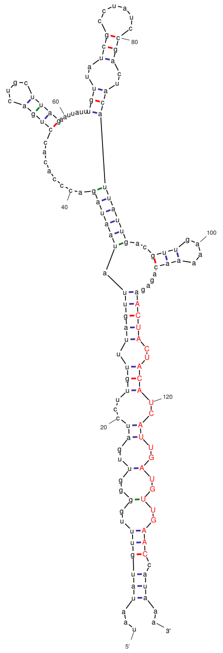

ptc-MIR480b_MI0002391_MIMAT0002097

Output of sir_graph (fl)
by D. Stewart and M. Zuker

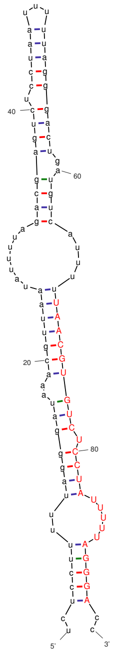

ptc-MIR478u_MI0002388_MIMAT0002094

Output of sir_graph (fl)
by D. Stewart and M. Zuker

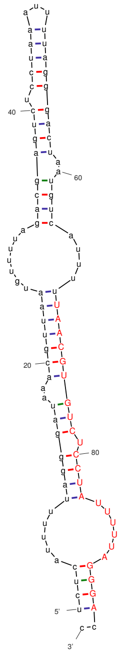

ptc-MIR478s_MI0002387_MIMAT0002093

Output of sir_graph (fl)
by D. Stewart and M. Zuker

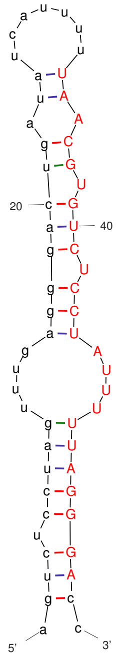

ptc-MIR478r_MI0002386_MIMAT0002092

Output of sir_graph (fl)
by D. Stewart and M. Zuker

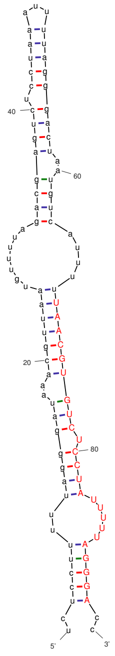

ptc-MIR478q_MI0002385_MIMAT0002091

Output of sir_graph (fl)
by D. Stewart and M. Zuker

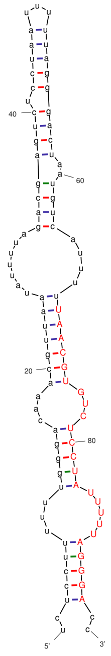

ptc-MIR478p_MI0002384_MIMAT0002090

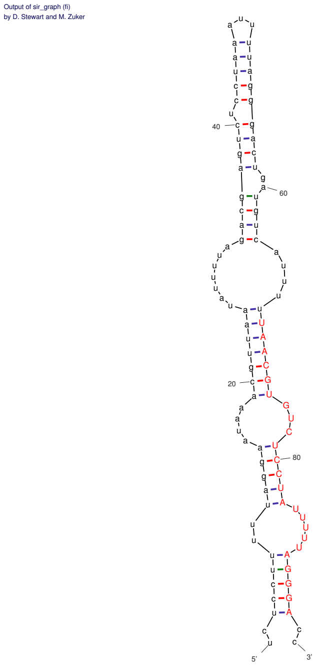

ptc-MIR478o_MI0002383_MIMAT0002089

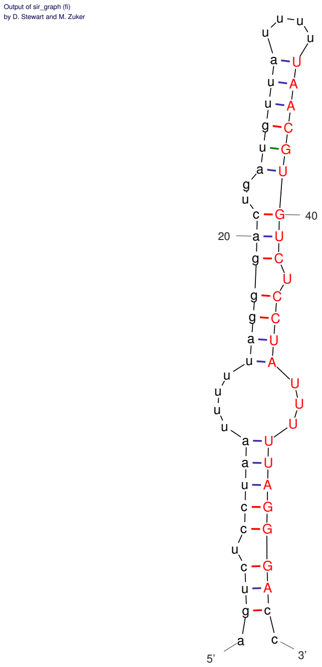

ptc-MIR478n_MI0002382_MIMAT0002088

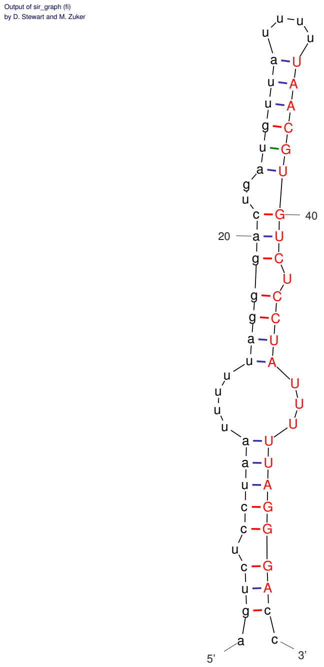

ptc-MIR478m_MI0002381_MIMAT0002087

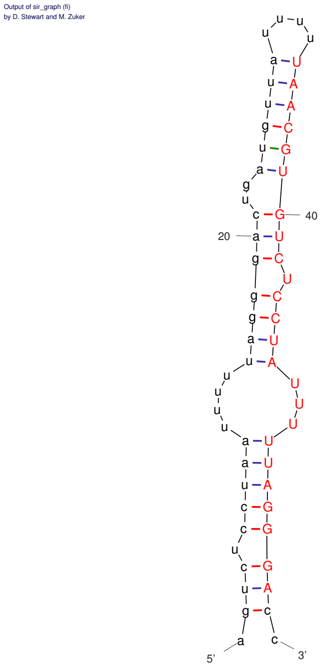

ptc-MIR478l_MI0002380_MIMAT0002086

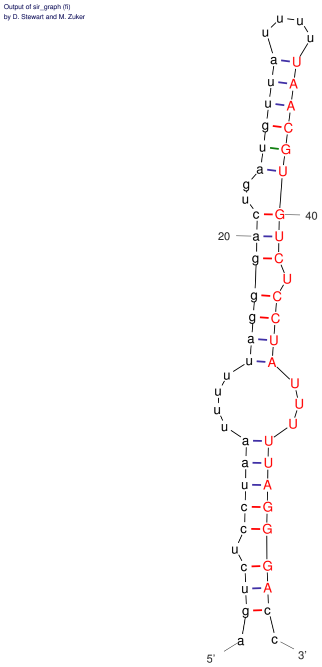

ptc-MIR478k_MI0002379_MIMAT0002085

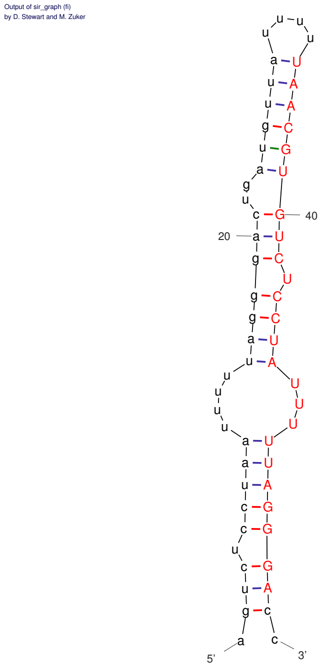

ptc-MIR478j_MI0002378_MIMAT0002084

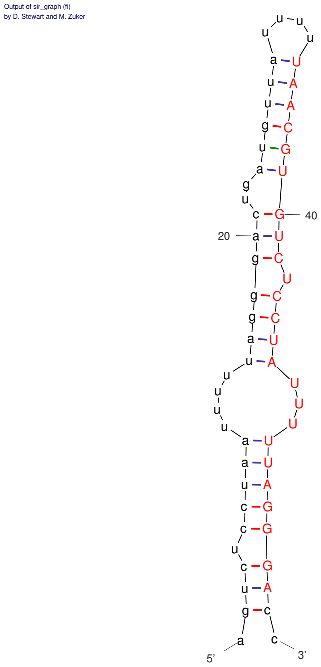

ptc-MIR478i_MI0002377_MIMAT0002083

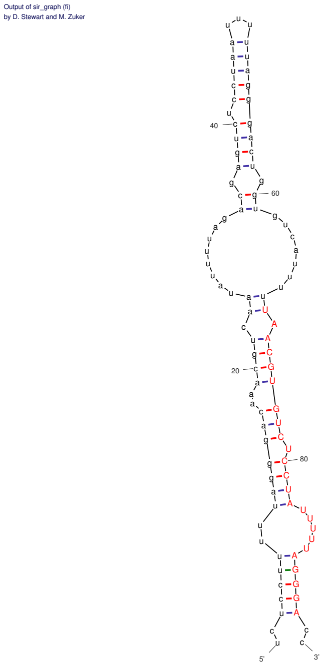

ptc-MIR478h_MI0002376_MIMAT0002082

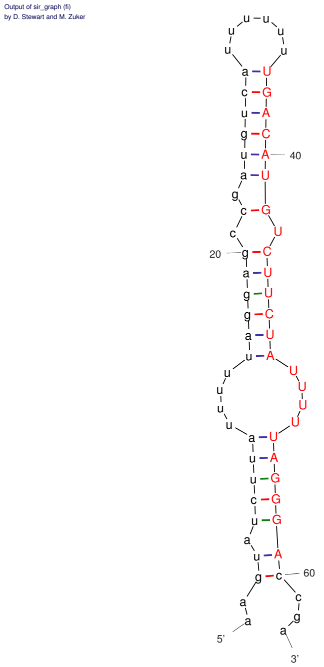

ptc-MIR478f_MI0002375_MIMAT0002081

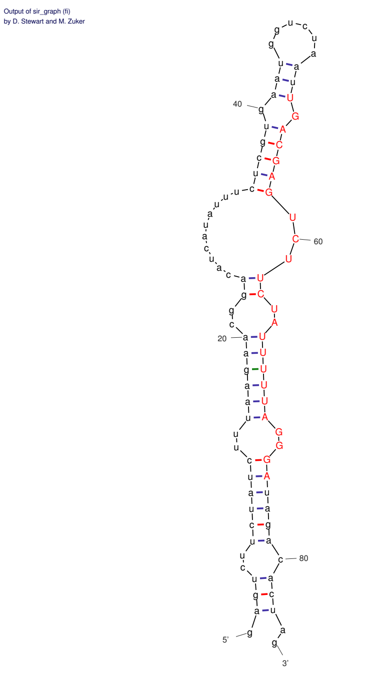

ptc-MIR478e_MI0002374_MIMAT0002080

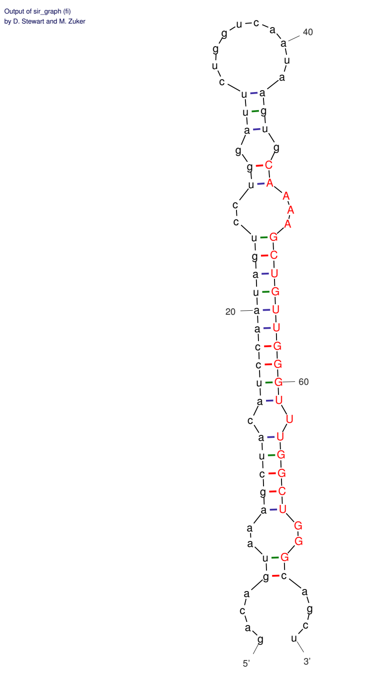

ptc-MIR474c_MI0002360_MIMAT0002066

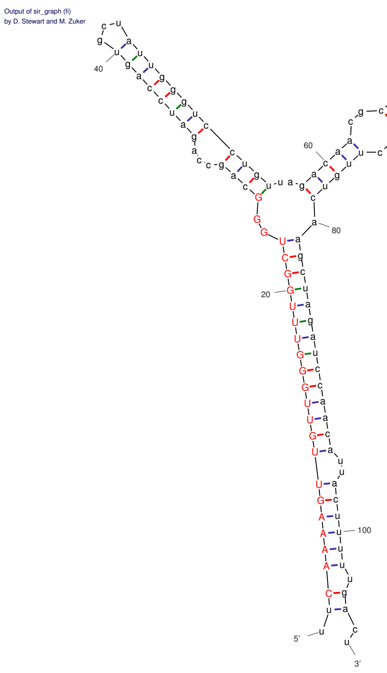

ptc-MIR474b_MI0002359_MIMAT0002065

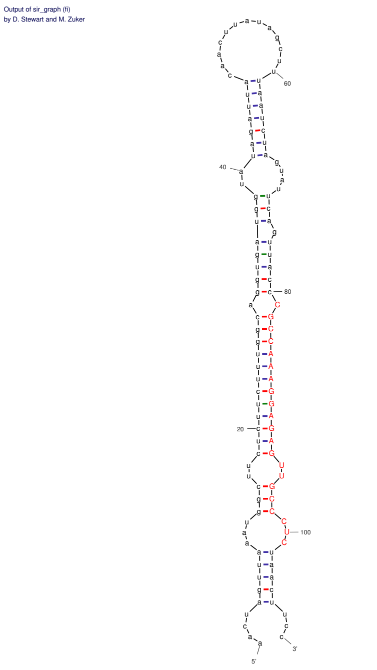

ptc-MIR399l_MI0002349_MIMAT0002055

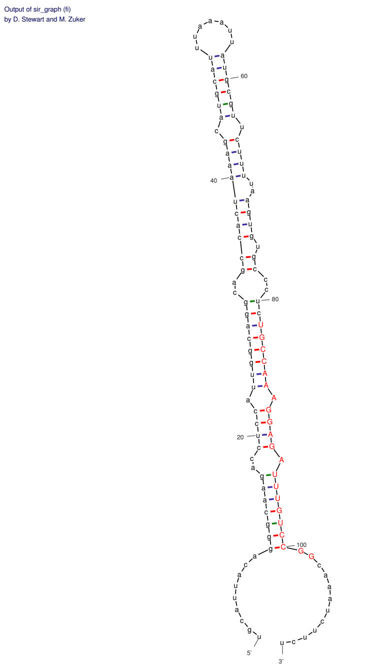

ptc-MIR399j_MI0002347_MIMAT0002053

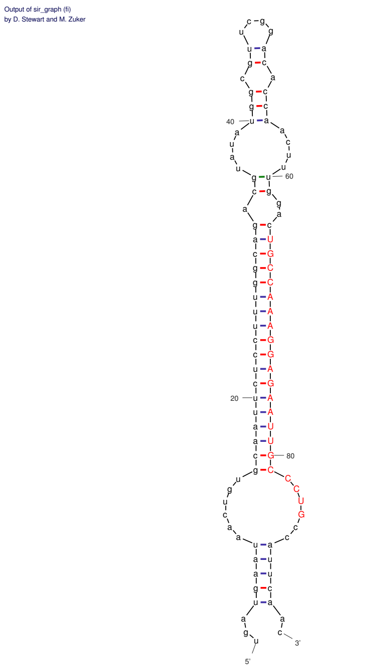

ptc-MIR399g_MI0002344_MIMAT0002050

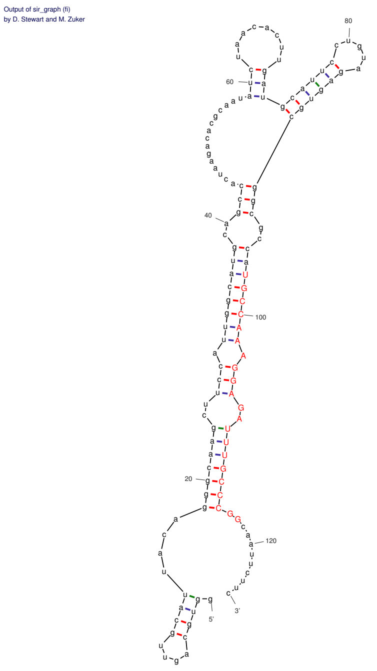

ptc-MIR399c_MI0002340_MIMAT0002046

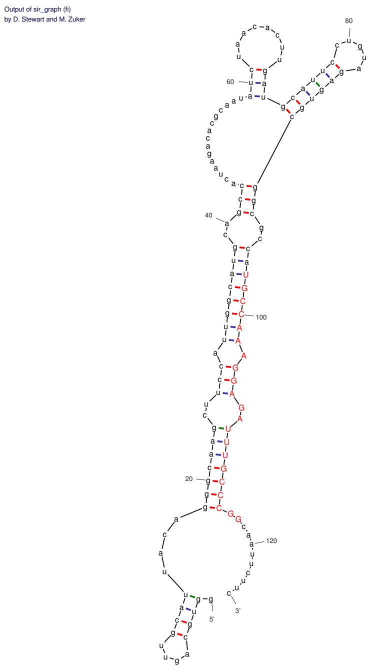

ptc-MIR399b_MI0002339_MIMAT0002045

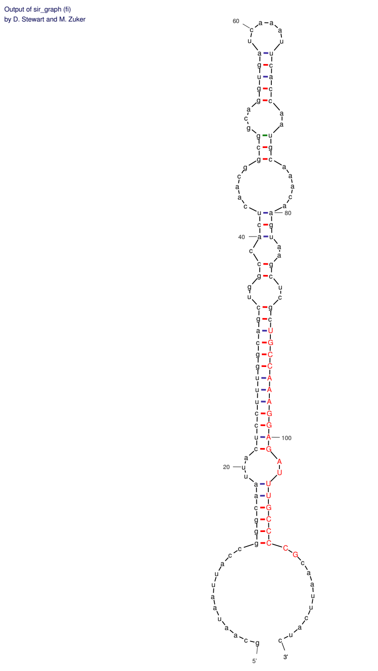

ptc-MIR399a_MI0002338_MIMAT0002044

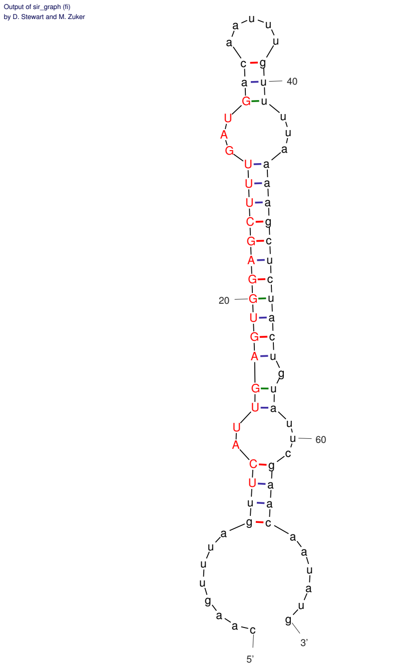

ptc-MIR397c_MI0002334_MIMAT0002040

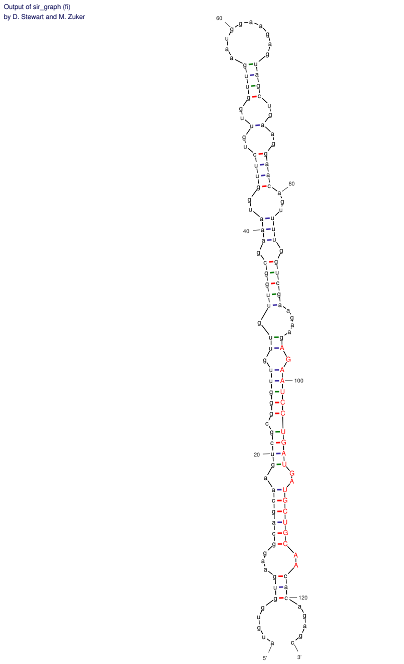

ptc-MIR172i_MI0002295_MIMAT0002001

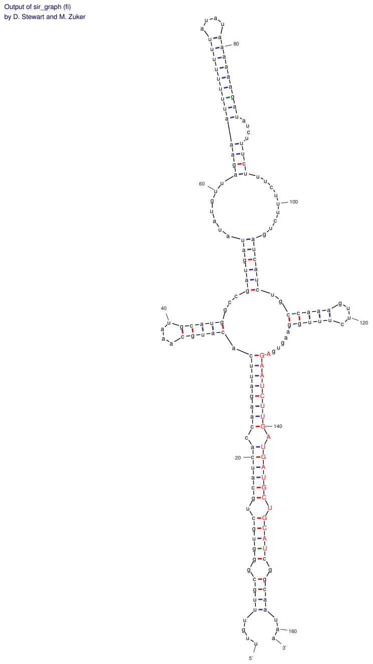

ptc-MIR172f_MI0002292_MIMAT0001998

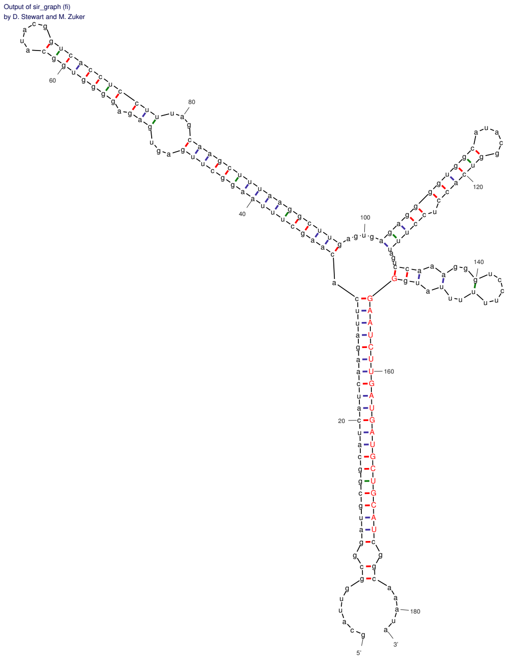

ptc-MIR172d_MI0002290_MIMAT0001996

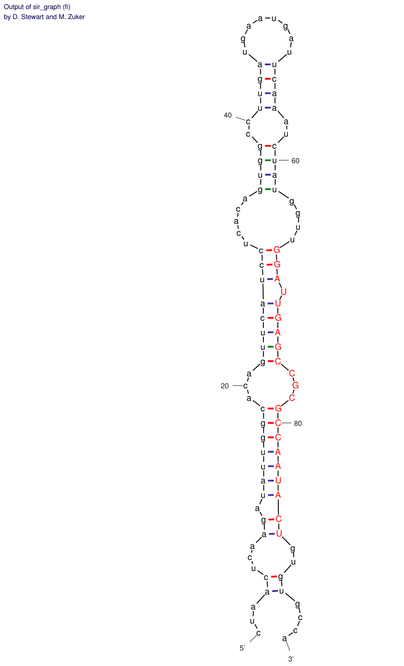

ptc-MIR171j_MI0002286_MIMAT0001992

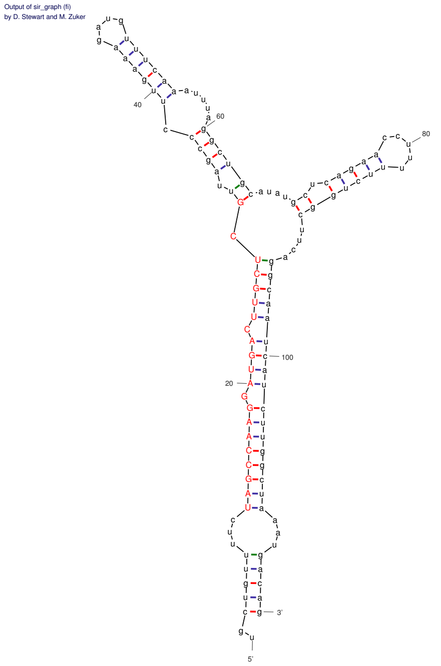

ptc-MIR169x_MI0002274_MIMAT0001980

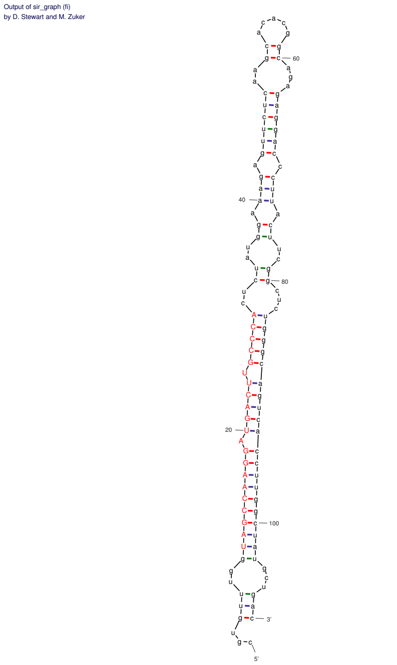

ptc-MIR169w_MI0002273_MIMAT0001979

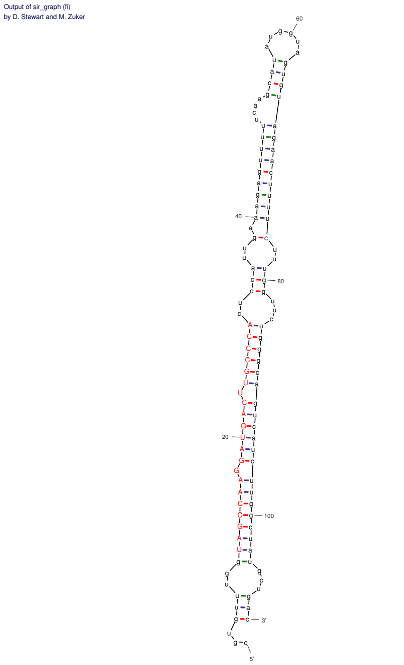

ptc-MIR169v_MI0002272_MIMAT0001978

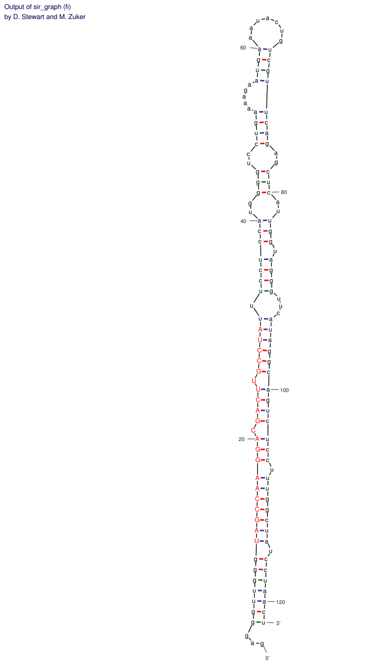

ptc-MIR169u_MI0002271_MIMAT0001977

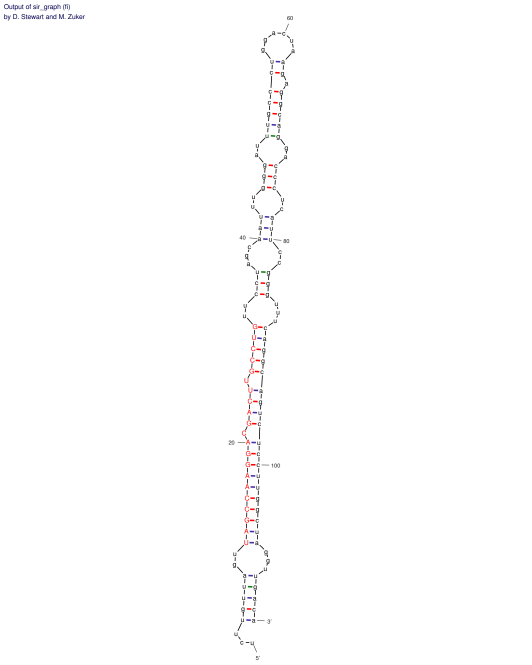

ptc-MIR169q_MI0002267_MIMAT0001973

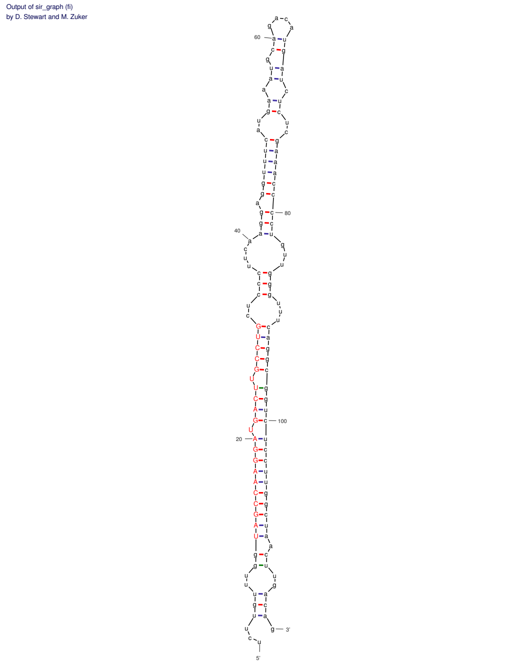

ptc-MIR169m_MI0002263_MIMAT0001969

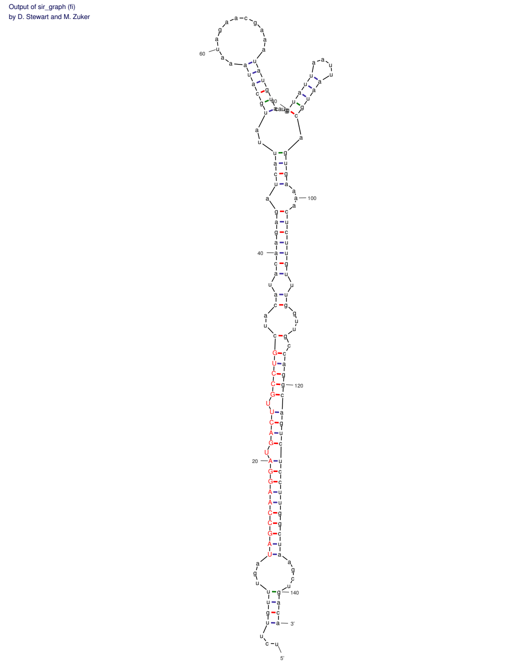

ptc-MIR169l_MI0002262_MIMAT0001968

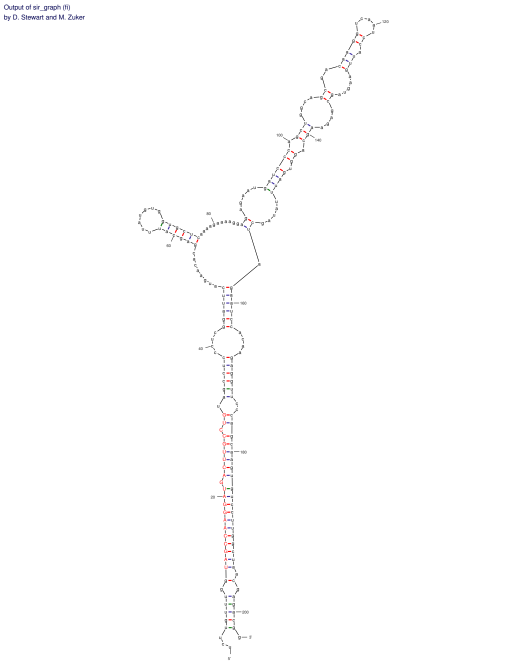

ptc-MIR169k_MI0002261_MIMAT0001967

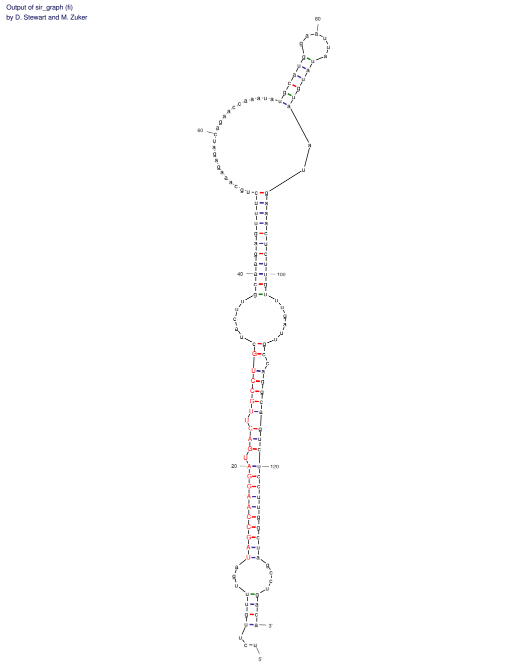

ptc-MIR169j_MI0002260_MIMAT0001966

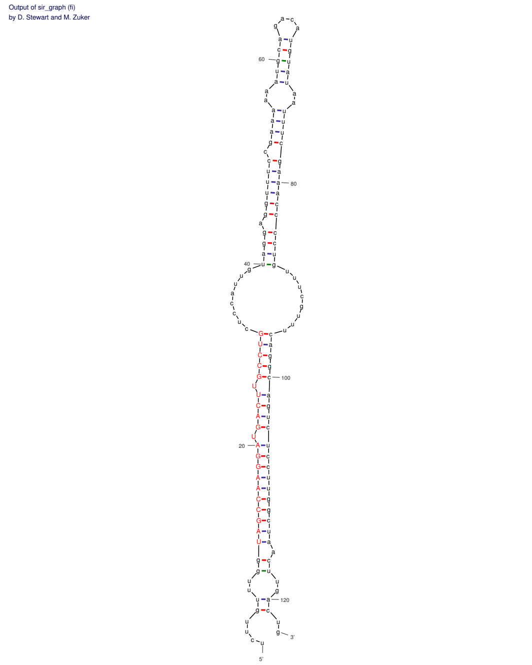

ptc-MIR169i_MI0002259_MIMAT0001965

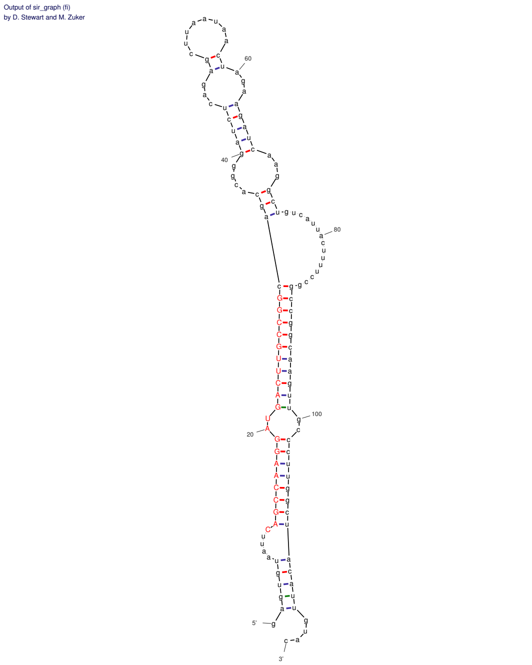

ptc-MIR169h_MI0002258_MIMAT0001964

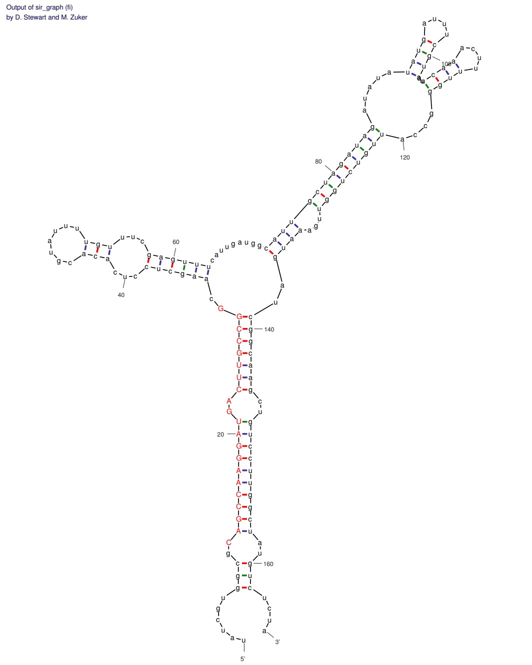

ptc-MIR169e_MI0002255_MIMAT0001961

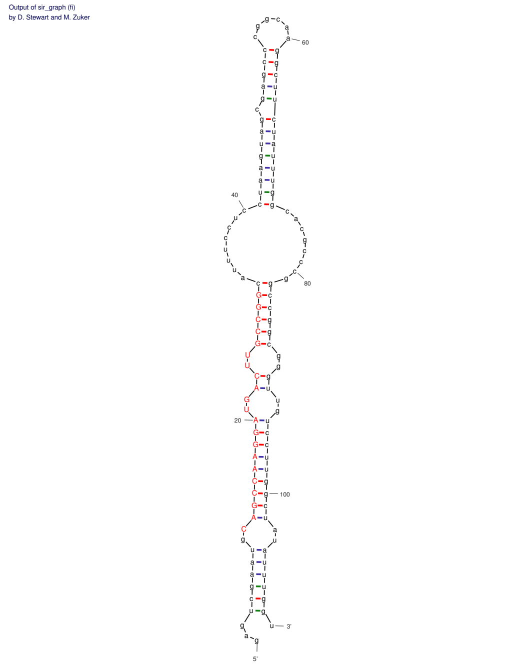

ptc-MIR169d_MI0002254_MIMAT0001960

Output of sir_graph (fi)
by D. Stewart and M. Zuker

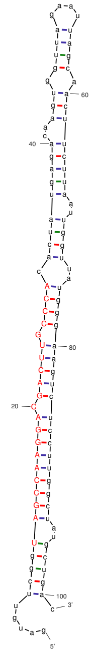

ptc-MIR169ad_MI0002249_MIMAT0001955

Output of sir_graph (fi)
by D. Stewart and M. Zuker

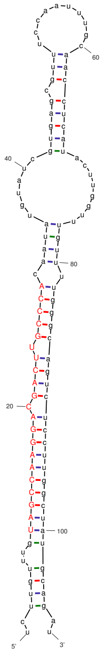

ptc-MIR169ac_MI0002248_MIMAT0001954

Output of sir_graph (fi)
by D. Stewart and M. Zuker

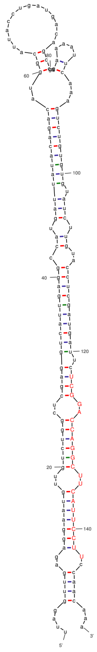

ptc-MIR166q_MI0002234_MIMAT0001940

Output of sir_graph (fi)
by D. Stewart and M. Zuker

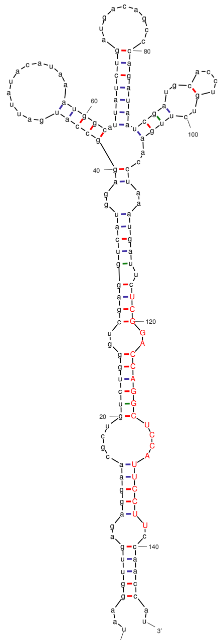

ptc-MIR166p_MI0002233_MIMAT0001939

Output of sir_graph (fi)
by D. Stewart and M. Zuker

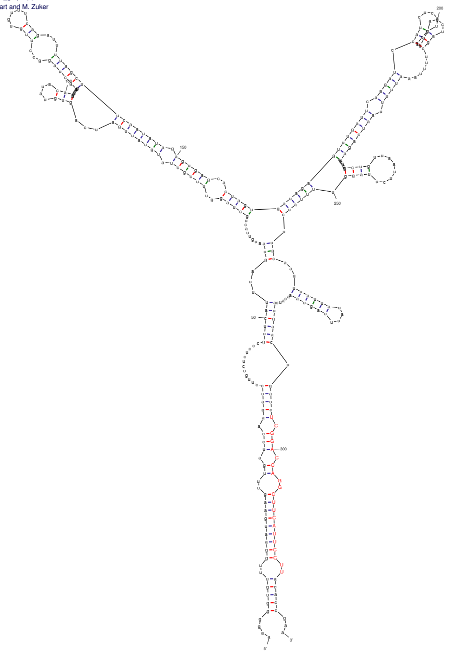

ptc-MIR166o_MI0002232_MIMAT0001938

Output of sir_graph (fi)
by D. Stewart and M. Zuker

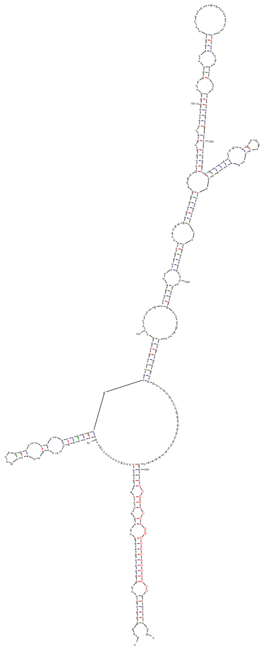

ptc-MIR166n_MI0002231_MIMAT0001937

Output of sir_graph (fi)
by D. Stewart and M. Zuker

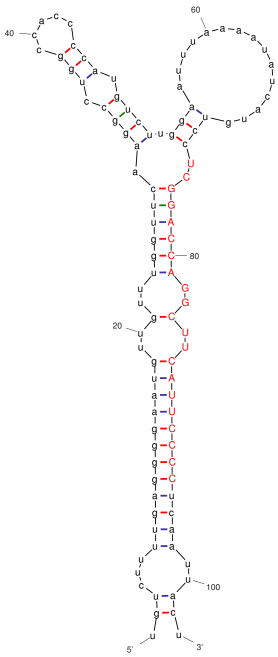

ptc-MIR166i_MI0002226_MIMAT0001932

Output of sir_graph (fi)
by D. Stewart and M. Zuker

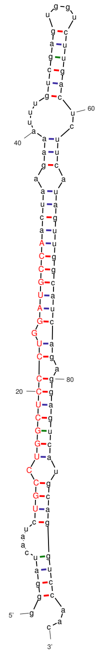

ptc-MIR160g_MI0002207_MIMAT0001913

Output of sir_graph (fi)
by D. Stewart and M. Zuker

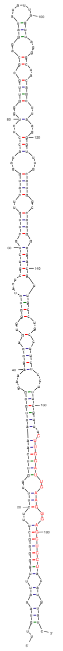

ptc-MIR159d_MI0002198_MIMAT0001904

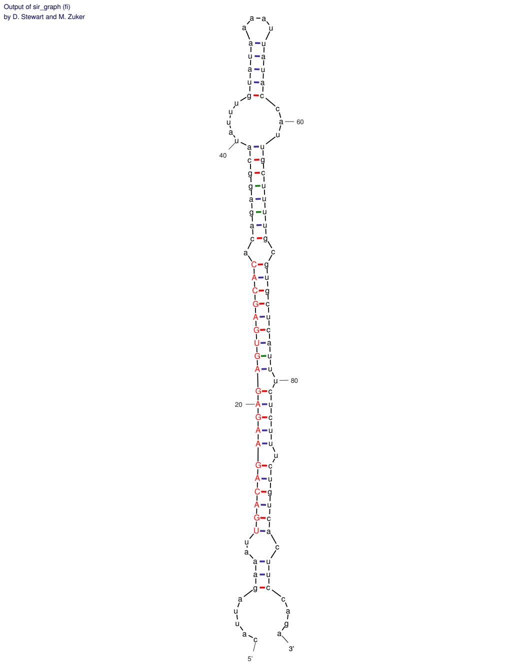

ptc-MIR156e_MI0002188_MIMAT0001894

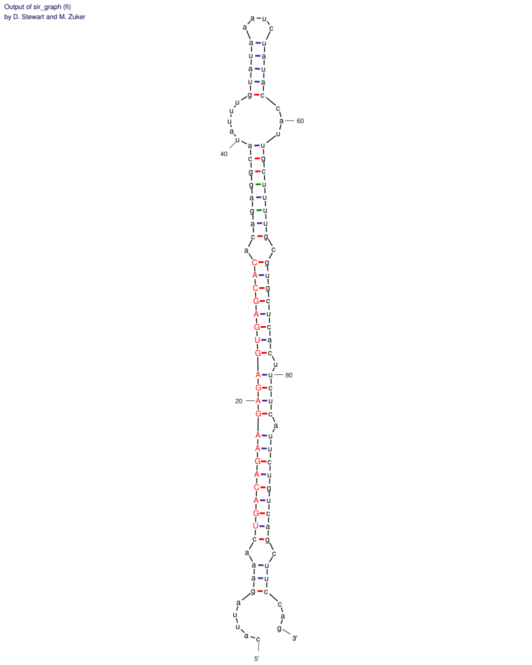

ptc-MIR156c_MI0002186_MIMAT0001892

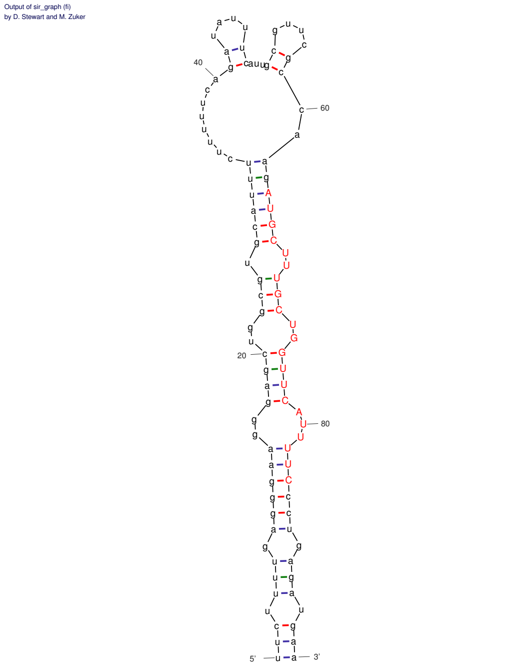

pta-MIR783_MI0005793_MIMAT0005004

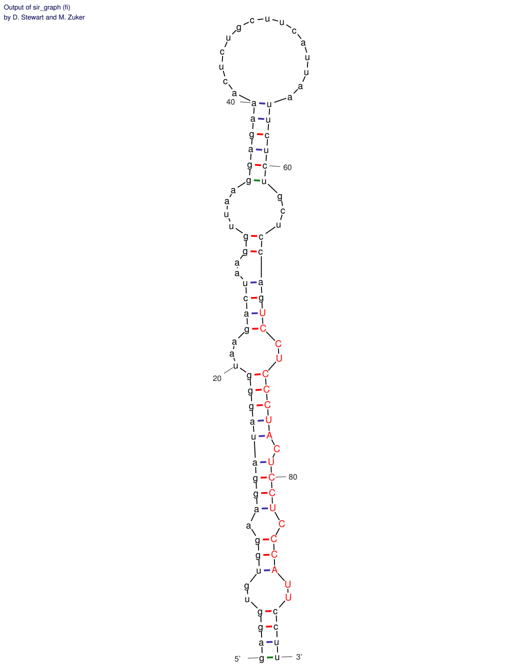

pta-MIR482d_MI0007053_MIMAT0006015

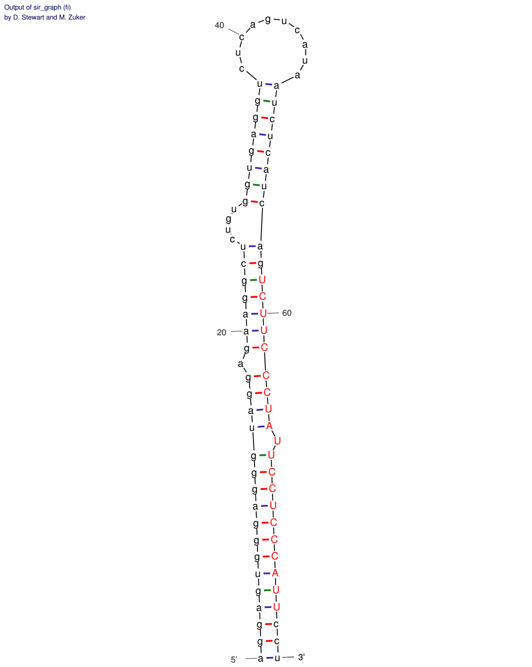

pta-MIR482c_MI0007052_MIMAT0006014

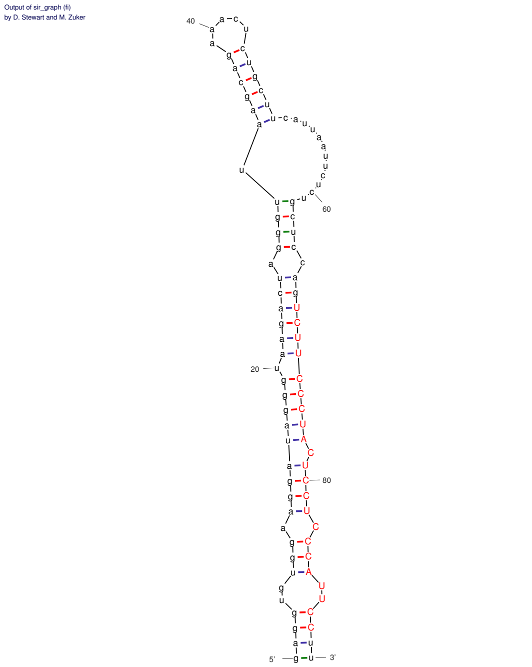

pta-MIR482b_MI0005792_MIMAT0005003

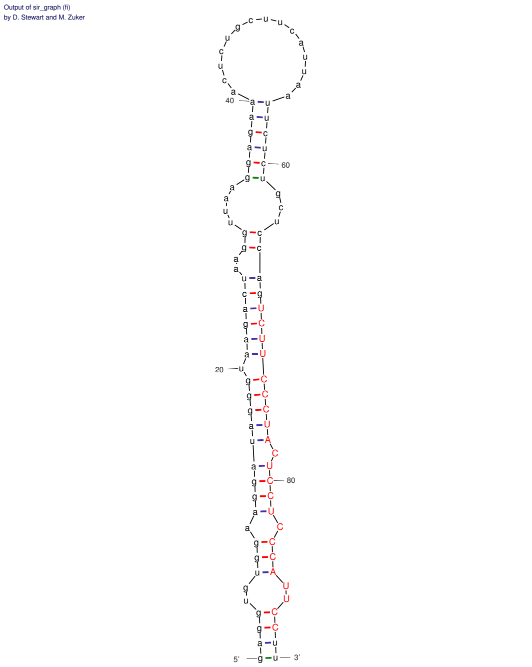

pta-MIR482a_MI0005791_MIMAT0005002

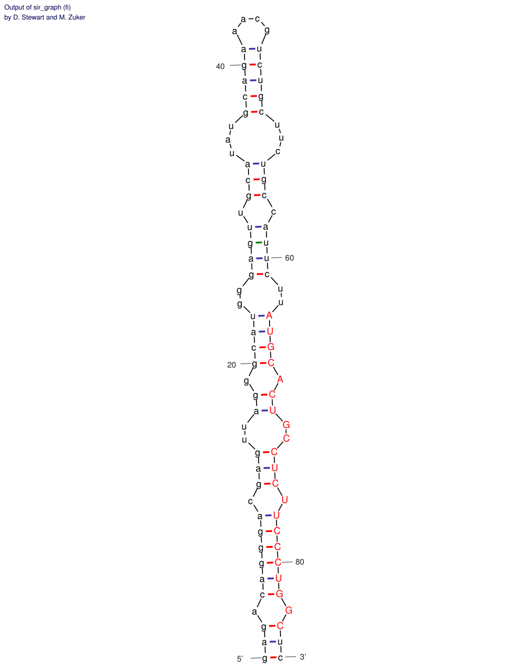

pta-MIR408_MI0005790_MIMAT0005001

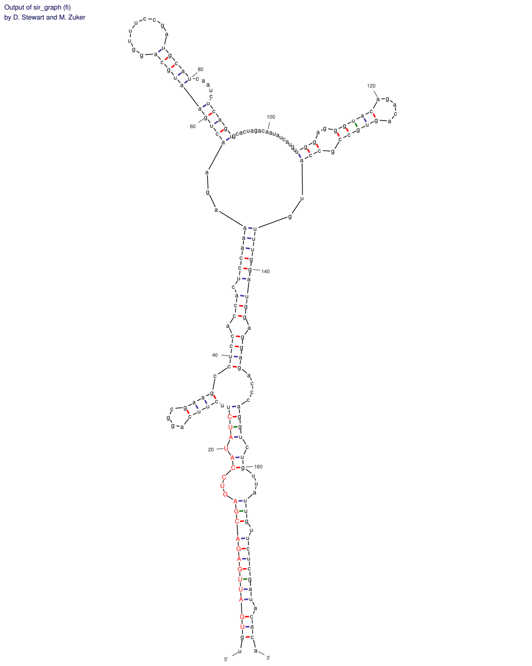

pta-MIR171_MI0005785_MIMAT0004996

Output of sir_graph (fi)
by D. Stewart and M. Zuker

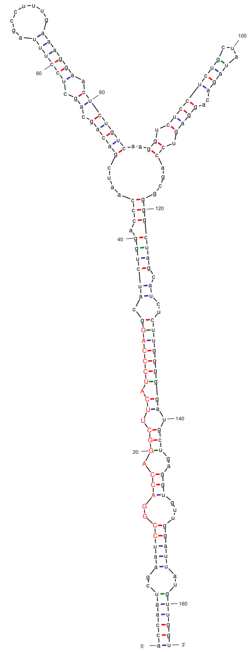

pta-MIR166c_MI0005784_MIMAT0004995

Output of sir_graph (fi)
by D. Stewart and M. Zuker

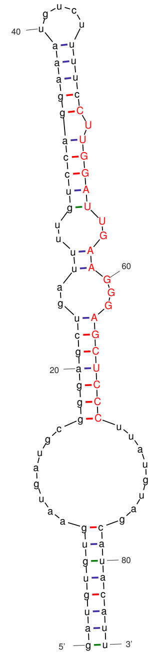

pta-MIR159c_MI0005780_MIMAT0004992

Output of sir_graph (fi)
by D. Stewart and M. Zuker

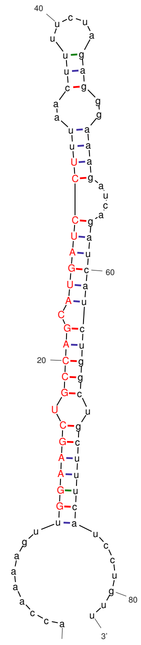

ppt-MIR167_MI0005661_MIMAT0004353

Output of sir_graph (fi)
by D. Stewart and M. Zuker

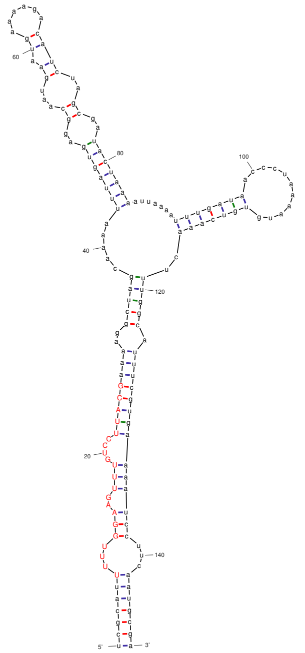

osa-MIR426_MI0001442_MIMAT0001338

Output of sir_graph (fi)
by D. Stewart and M. Zuker

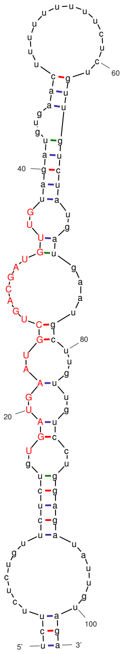

osa-MIR419_MI0001439_MIMAT0001335

Output of sir_graph (fi)
by D. Stewart and M. Zuker

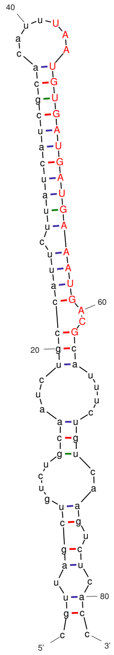

osa-MIR418_MI0001438_MIMAT0001334

Output of sir_graph (fi)
by D. Stewart and M. Zuker

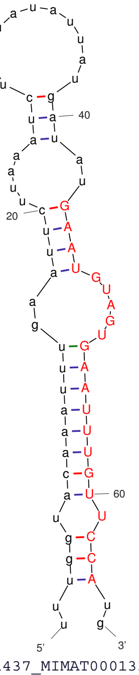

osa-MIR417_MI0001437_MIMAT0001333

Output of sir_graph (fi)
by D. Stewart and M. Zuker

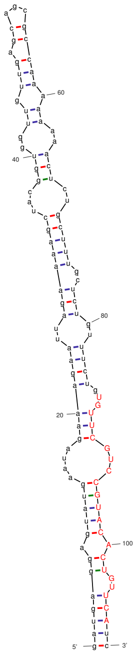

osa-MIR416_MI0001436_MIMAT0001332

Output of sir_graph (fi)
by D. Stewart and M. Zuker

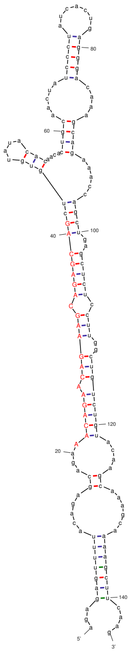

osa-MIR415_MI0001435_MIMAT0001331

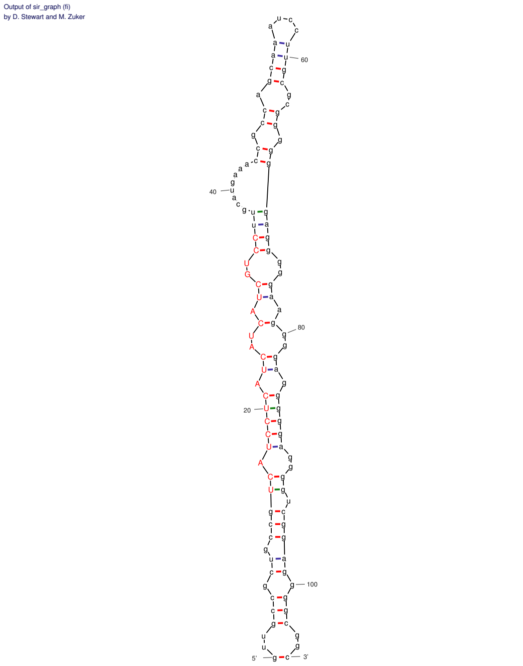

osa-MIR414_MI0001434_MIMAT0001330

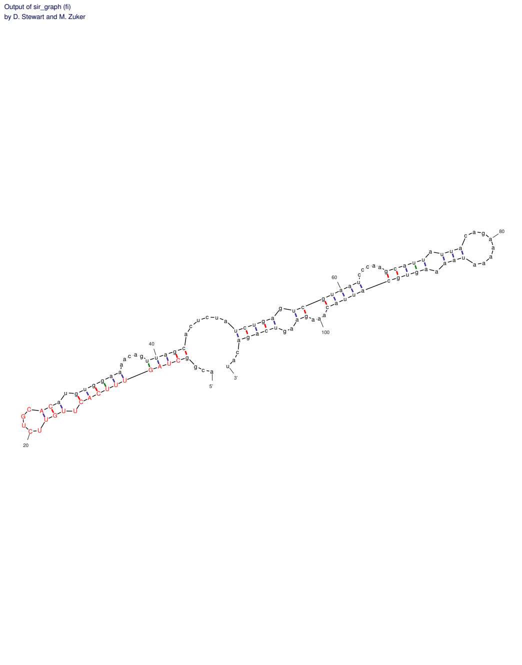

osa-MIR413_MI0001433_MIMAT0001329

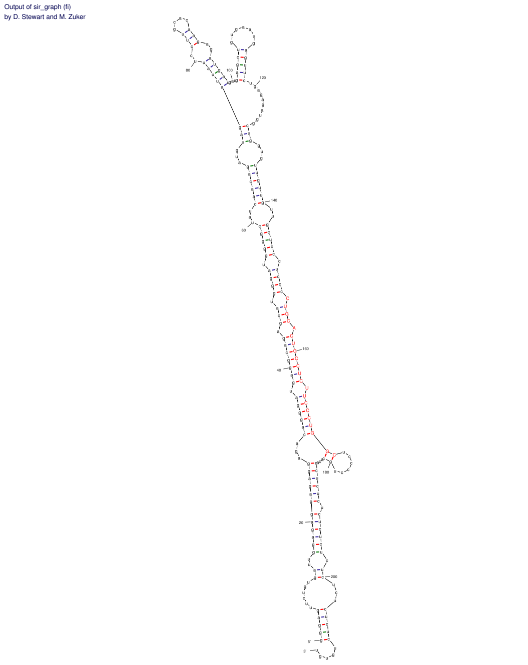

osa-MIR408_MI0001149_MIMAT0001079

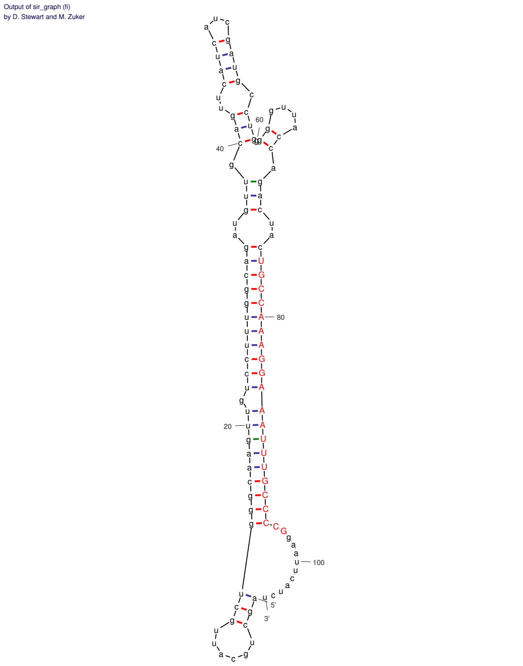

osa-MIR399k_MI0001063_MIMAT0000994

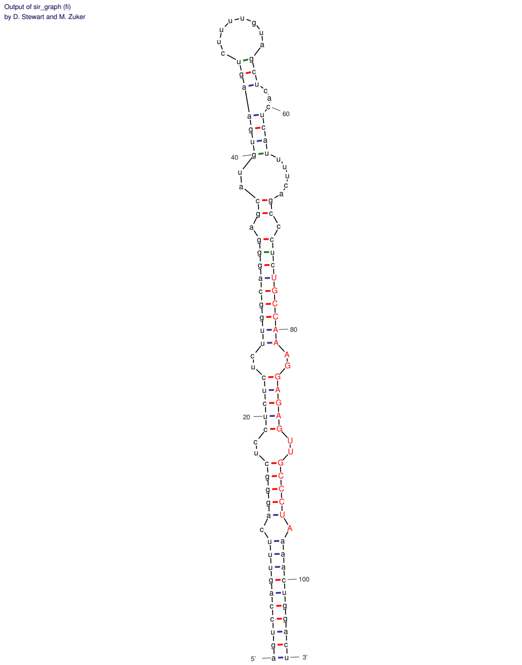

osa-MIR399j_MI0001062_MIMAT0000993

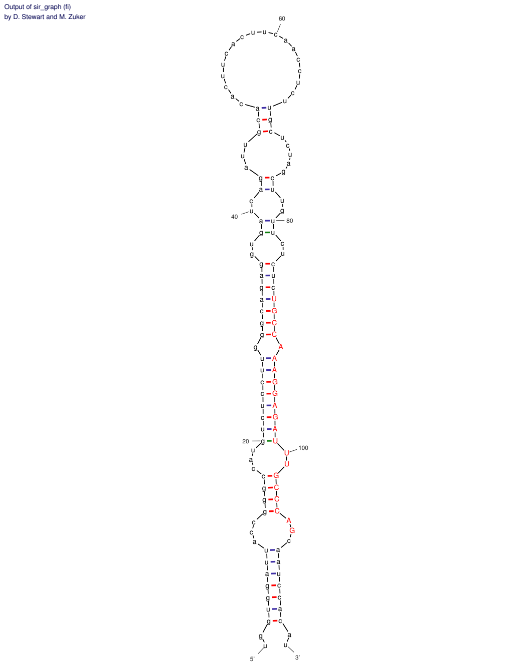

osa-MIR399f_MI0001058_MIMAT0000989

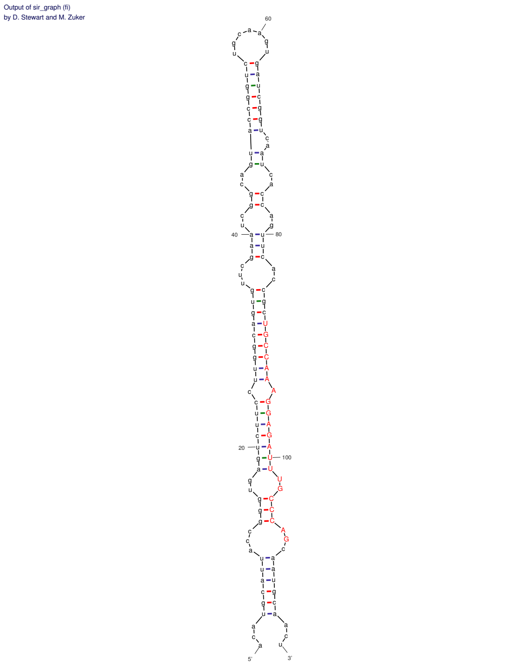

osa-MIR399e_MI0001057_MIMAT0000988

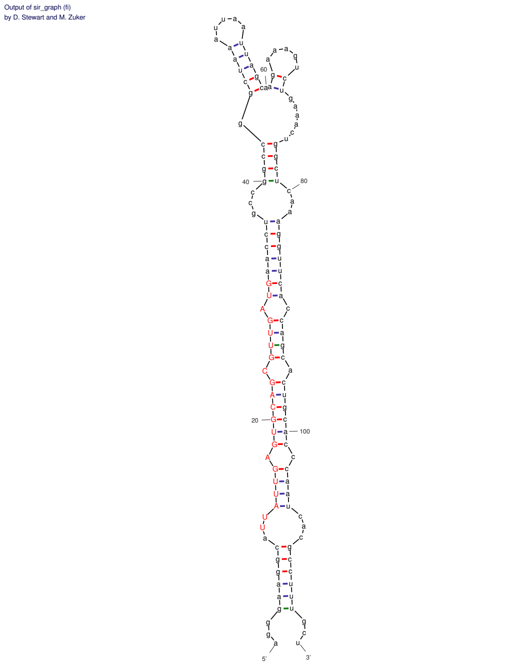

osa-MIR397b_MI0001050_MIMAT0000981

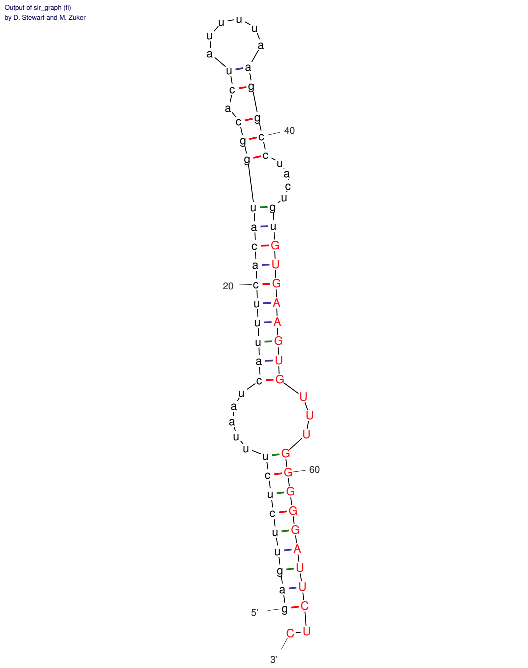

osa-MIR395w_MI0005091_MIMAT0003877

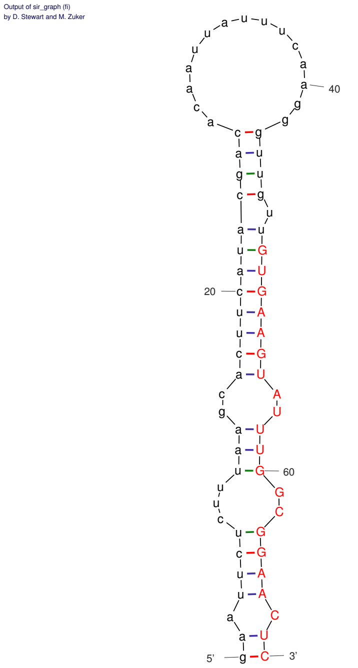

osa-MIR395v_MI0005090_MIMAT0003876

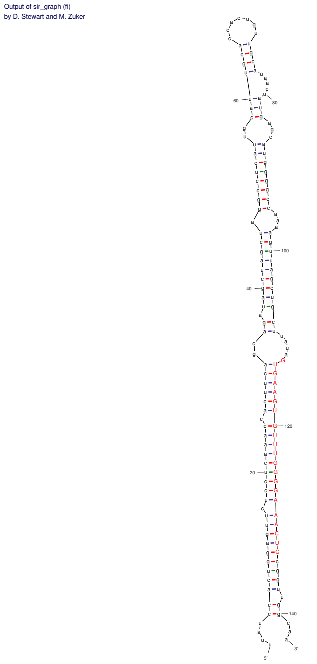

osa-MIR395t_MI0001038_MIMAT0000969

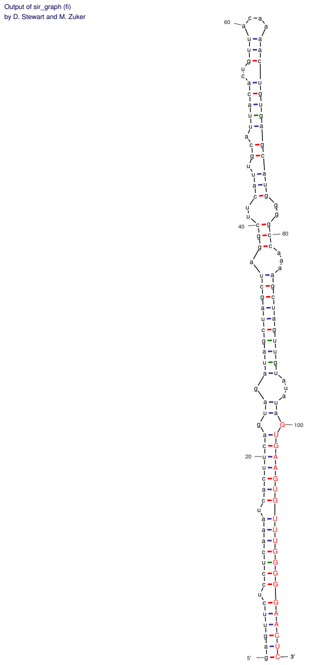

osa-MIR395m_MI0005084_MIMAT0003870

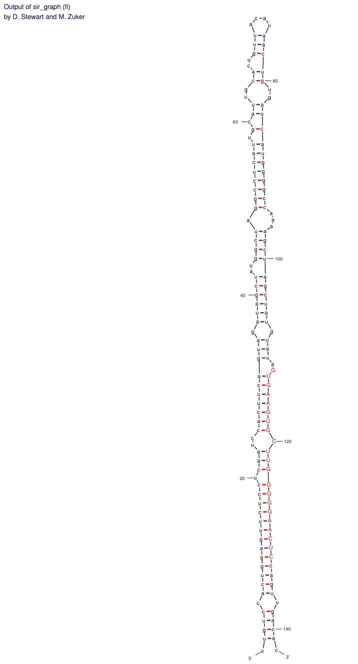

osa-MIR395a_MI0001042_MIMAT0000973

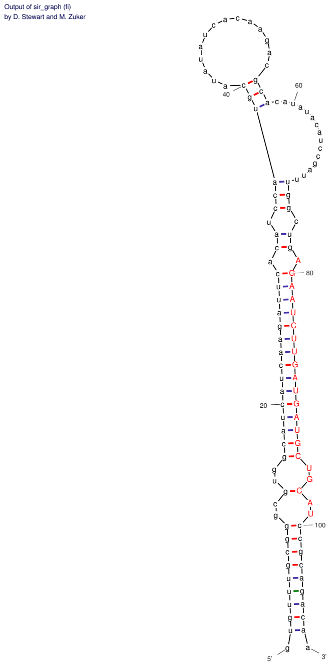

osa-MIR172a_MI0001139_MIMAT0001069

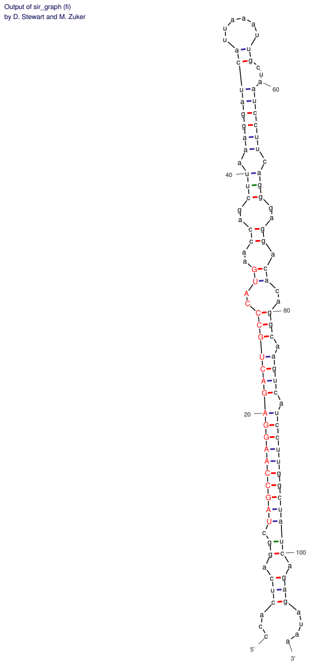

osa-MIR169q_MI0001132_MIMAT0001062

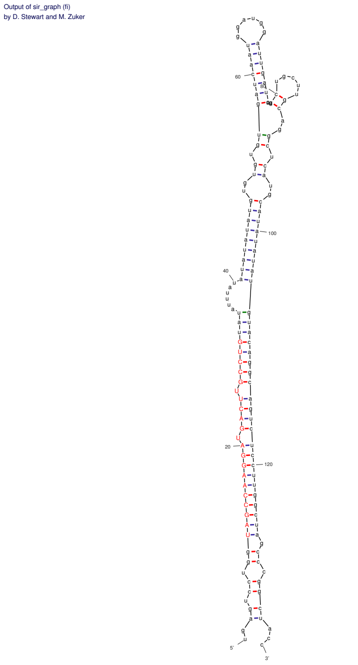

osa-MIR169m_MI0001128_MIMAT0001058

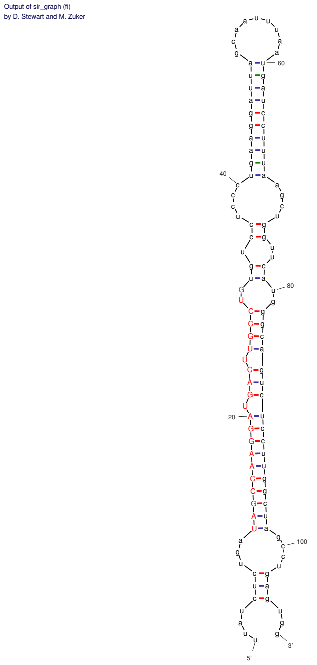

osa-MIR169l_MI0001127_MIMAT0001057

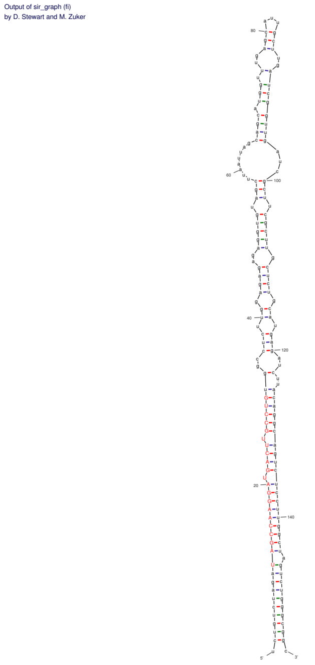

osa-MIR169k_MI0001126_MIMAT0001056

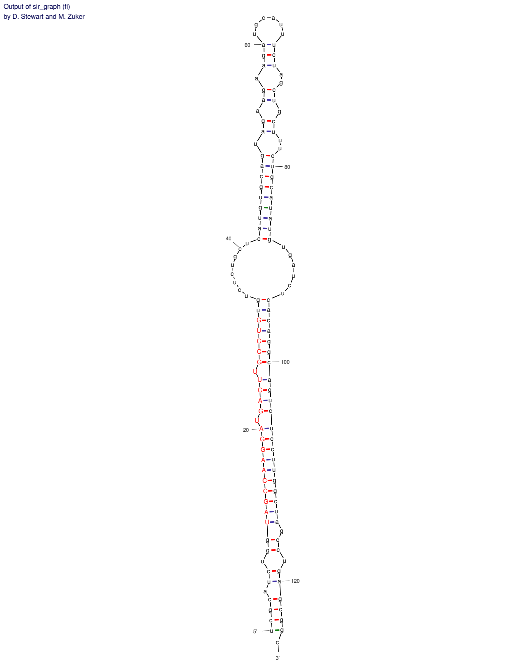

osa-MIR169j_MI0001125_MIMAT0001055

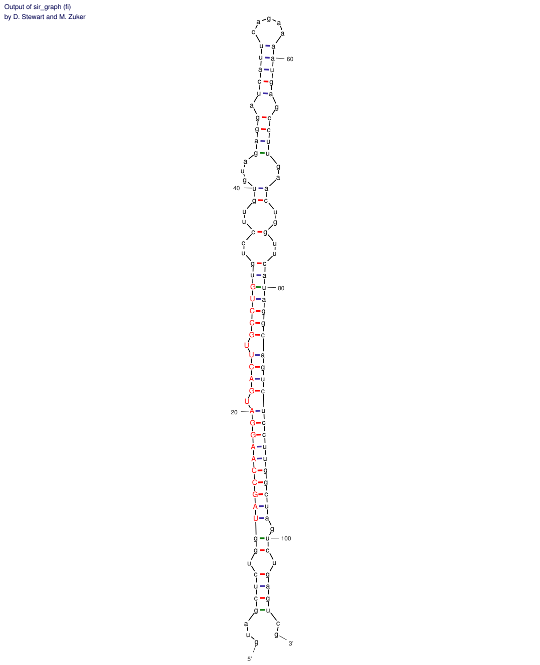

osa-MIR169i_MI0001124_MIMAT0001054

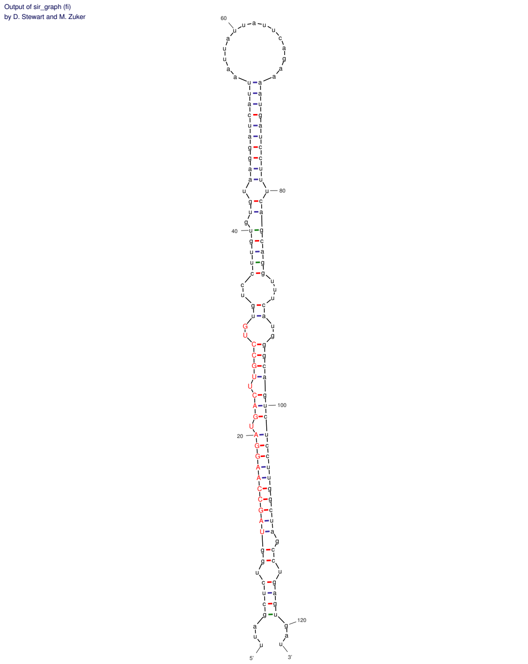

osa-MIR169h_MI0001123_MIMAT0001053

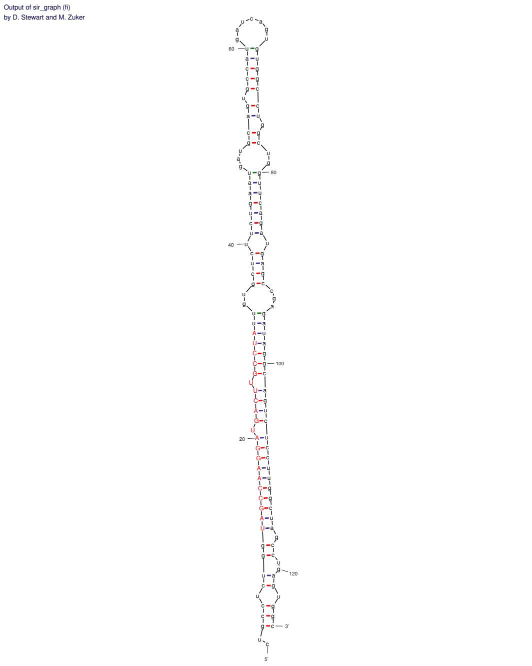

osa-MIR169g_MI0001122_MIMAT0001052

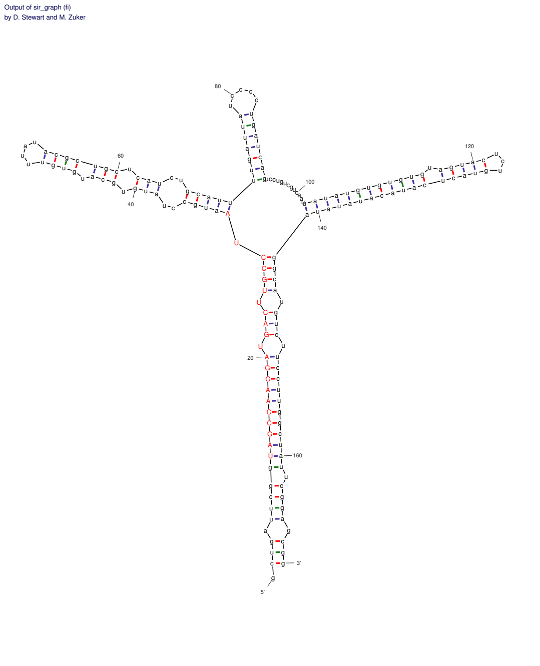

osa-MIR169f_MI0001121_MIMAT0001051

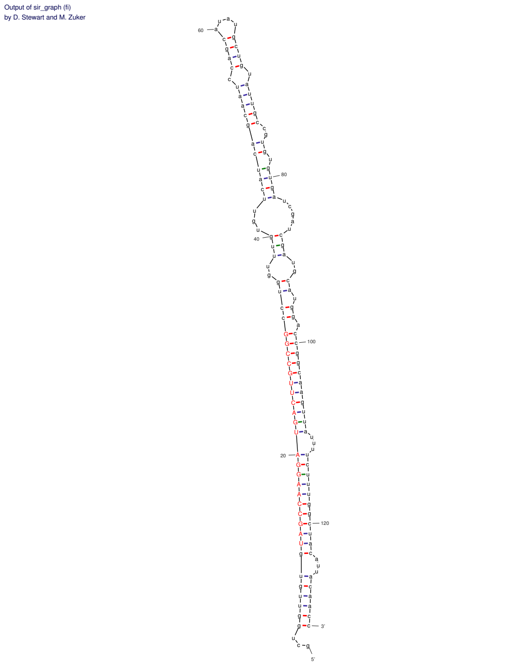

osa-MIR169e_MI0001120_MIMAT0001050

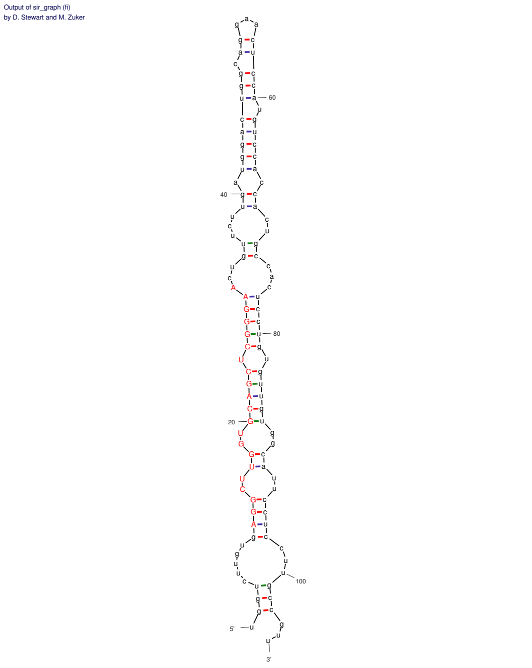

osa-MIR168b_MI0001116_MIMAT0001046

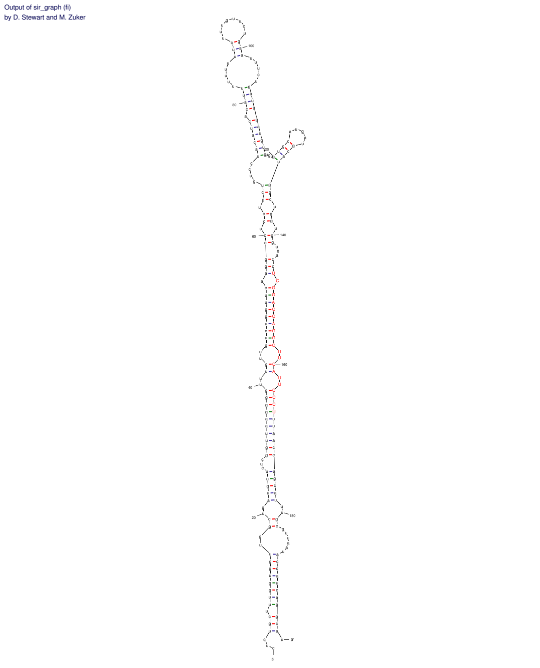

osa-MIR166m_MI0001157_MIMAT0001087

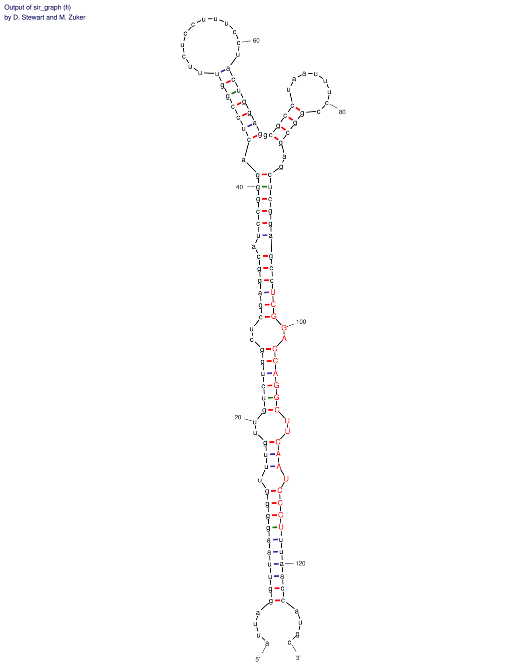

osa-MIR166k_MI0001107_MIMAT0001037

Output of sir_graph (fi)
by D. Stewart and M. Zuker

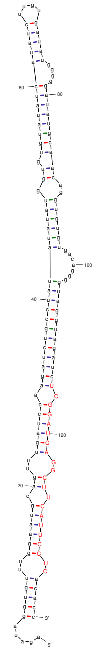

osa-MIR166j_MI0001106_MIMAT0001036

Output of sir_graph (fi)
by D. Stewart and M. Zuker

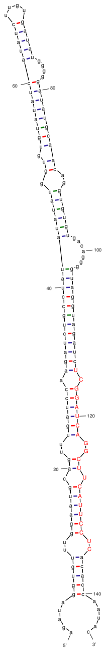

osa-MIR166i_MI0001144_MIMAT0001074

Output of sir_graph (fi)
by D. Stewart and M. Zuker

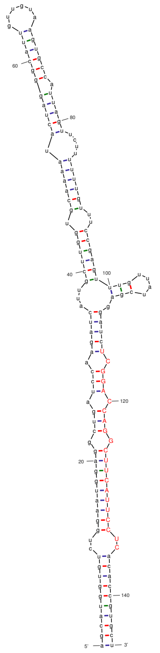

osa-MIR166g_MI0001142_MIMAT0001072

Output of sir_graph (fi)
by D. Stewart and M. Zuker

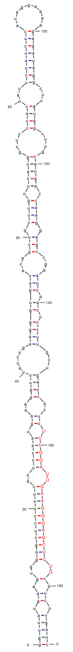

osa-MIR159b_MI0001093_MIMAT0001023

Output of sir_graph (fi)
by D. Stewart and M. Zuker

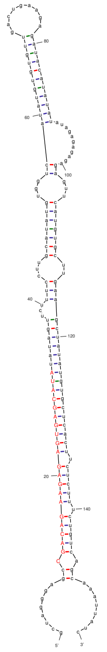

osa-MIR156l_MI0001091_MIMAT0001021

Output of sir_graph (fi)
by D. Stewart and M. Zuker

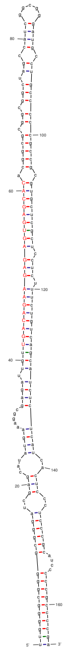

osa-MIR156j_MI0000662_MIMAT0000627

Output of sir_graph (fi)
by D. Stewart and M. Zuker

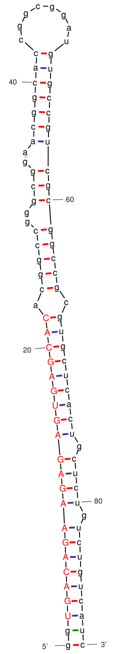

osa-MIR156i_MI0000661_MIMAT0000626

Output of sir_graph (fi)
by D. Stewart and M. Zuker

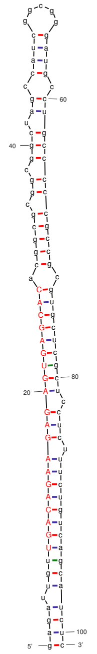

osa-MIR156h_MI0000660_MIMAT0000625

Output of sir_graph (fi)
by D. Stewart and M. Zuker

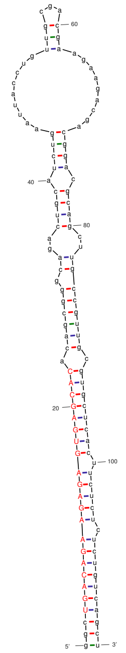

osa-MIR156g_MI0000659_MIMAT0000624

Output of sir_graph (fl)
by D. Stewart and M. Zuker

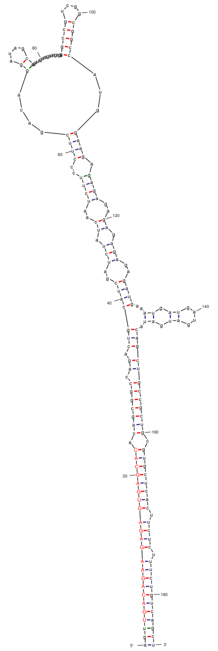

osa-MIR156f_MI0000658_MIMAT0000623

Output of sir_graph (fl)
by D. Stewart and M. Zuker

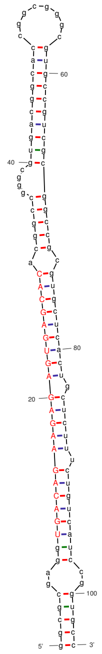

osa-MIR156e_MI0000657_MIMAT0000622

Output of sir_graph (fl)
by D. Stewart and M. Zuker

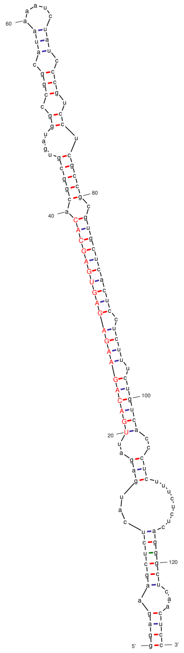

osa-MIR156d_MI0000656_MIMAT0000621

Output of sir_graph (fl)
by D. Stewart and M. Zuker

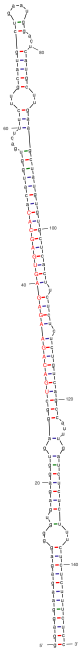

osa-MIR156c_MI0000655_MIMAT0000620

Output of sir_graph (fl)
by D. Stewart and M. Zuker

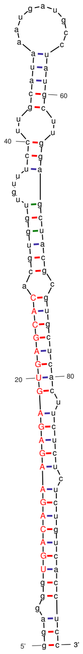

osa-MIR156a_MI0000653_MIMAT0000618

Output of sir_graph (fl)
by D. Stewart and M. Zuker

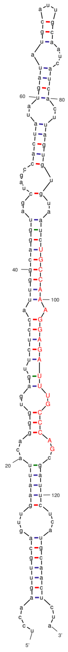

mtr-MIR399e_MI0001750_MIMAT0001652

Output of sir_graph (fl)
by D. Stewart and M. Zuker

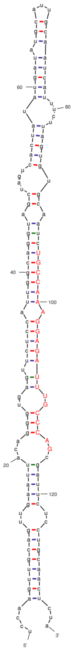

mtr-MIR399a_MI0001749_MIMAT0001651

Output of sir_graph (fl)
by D. Stewart and M. Zuker

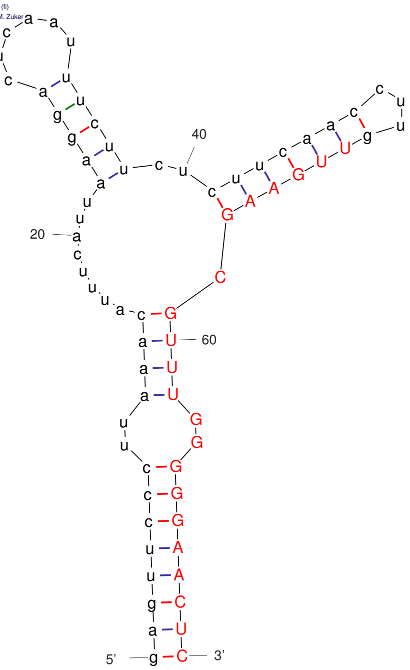

mtr-MIR395p_MI0005083_MIMAT0003869

Output of sir_graph (fl)
by D. Stewart and M. Zuker

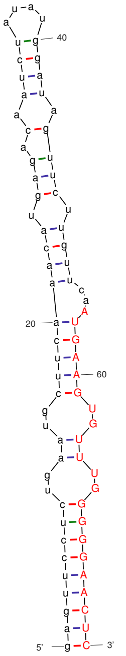

mtr-MIR395j_MI0005077_MIMAT0003863

Output of sir_graph (©)
by D. Stewart and M. Zuker

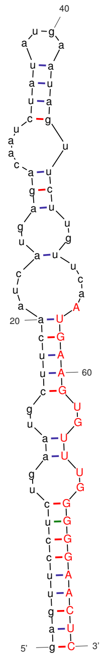

mtr-MIR395i_MI0005076_MIMAT0003862

Output of sir_graph (©)
by D. Stewart and M. Zuker

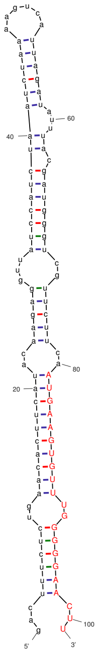

mtr-MIR395h_MI0005075_MIMAT0003861

Output of sir_graph (©)
by D. Stewart and M. Zuker

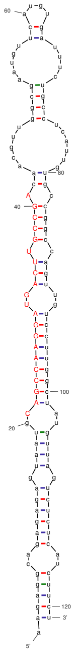

mtr-MIR169b_MI0001742_MIMAT0001644
