# Supplementary material for: MicroPC (μPC): A comprehensive resource for predicting and comparing plant microRNAs
Source: BMC Genomics. 2009 Aug 7;10:366. doi: 10.1186/1471-2164-10-366 (PMC2907689; doi:10.1186/1471-2164-10-366)
Supplement: Additional file 3 — List of plant species and their EST sequences obtained from PlantGDB (November, 2008) [file 1471-2164-10-366-S3.pdf]

List of plant species and their EST sequences obtained from PlantGDB (November, 2008).

No.	Plant name	Total EST sequences
1	*Actinidia chinensis*	18734
2	*Actinidia deliciosa*	24981
3	*Actinidia eriantha*	5282
4	*Adiantum capillus veneris*	7948
5	*Allium cepa*	12990
6	*Amborella trichopoda*	15772
7	*Antirrhinum majus*	13801
8	*Aquilegia formosa x Aquilegia pubescens*	19615
9	*Arabidopsis thaliana*	324630
10	*Arachis hypogaea*	9236
11	*Aristolochia fimbriata*	7967
12	*Artemisia annua*	24963
13	*Avena sativa*	5597
14	*Beta vulgaris*	18009
15	*Brachypodium distachyon*	9924
16	*Brassica napus*	131259
17	*Brassica oleracea*	12843
18	*Brassica oleracea var alboglabra*	9135
19	*Brassica rapa*	43468
20	*Bruguiera gymnorhiza*	7160
21	*Capsicum annuum*	15278
22	*Carica papaya*	42169
23	*Carthamus tinctorius*	22428
24	*Catharanthus roseus*	9675
25	*Cenchrus ciliaris*	12364
26	*Centaurea maculosa*	28506
27	*Centaurea solstitialis*	26288
28	*Ceratopteris richardii*	4234
29	*Chlamydomonas reinhardtii*	50380
30	*Cichorium endivia*	21603
31	*Cichorium intybus*	25368
32	*Citrus aurantium*	11427
33	*Citrus clementina*	37350
34	*Citrus reticulata*	46876
35	*Citrus sinensis*	105294
36	*Citrus x limonia*	9857
37	*Coffea arabica*	1093
38	*Coffea canephora*	20168
39	*Cryptomeria japonica*	24299
40	*Cucumis melo subsp melo*	13692
41	*Cucumis sativus*	3954
42	*Curcuma longa*	6627
43	*Cyamopsis tetragonoloba*	7618
44	*Cycas rumphii*	10901
45	*Elaeis guineensis*	11420
46	*Eragrostis curvula*	10948
47	*Eucalyptus globulus*	5885
48	*Eucalyptus gunnii*	8171
49	*Euphorbia esula*	27994
50	*Festuca arundinacea*	21517

List of plant species and their EST sequences obtained from PlantGDB (November, 2008) (cont.)

No.	Plant name	Total EST sequences
51	*Fragaria vesca*	11527
52	*Gerbera hybrid cv Terra Regina*	9018
53	*Ginkgo biloba*	8819
54	*Glycine max*	182383
55	*Glycine soja*	11332
56	*Gnetum gnemon*	6193
57	*Gossypium*	98639
58	*Gossypium arboreum*	24597
59	*Gossypium hirsutum*	63138
60	*Gossypium raimondii*	27355
61	*Helianthus annuus*	40695
62	*Helianthus argophyllus*	20511
63	*Helianthus ciliaris*	16353
64	*Helianthus exilis*	21776
65	*Helianthus paradoxus*	6398
66	*Helianthus petiolaris*	14885
67	*Helianthus tuberosus*	25708
68	*Hevea brasiliensis*	3849
69	*Hordeum vulgare*	102435
70	*Humulus lupulus*	4824
71	*Ipomoea batatas*	8808
72	*Ipomoea nil*	22947
73	*Juglans hindsii x Juglans regia*	7753
74	*Lactuca perennis*	12747
75	*Lactuca saligna*	12186
76	*Lactuca sativa*	29977
77	*Lactuca serriola*	22957
78	*Lactuca virosa*	12839
79	*Leymus cinereus x Leymus triticoides*	13567
80	*Limnanthes alba*	4359
81	*Linum usitatissimum*	7921
82	*Liriodendron tulipifera*	14232
83	*Lotus corniculatus*	39940
84	*Lotus japonicus*	43596
85	*Lycopersicon esculentum*	40951
86	*Lycopersicon hirsutum*	4031
87	*Lycopersicon pennellii*	3716
88	*Malus x domestica*	71246
89	*Manihot esculenta*	30401
90	*Marchantia polymorpha*	10959
91	*Medicago sativa*	6104
92	*Medicago truncatula*	57231
93	*Mesembryanthemum crystallinum*	11317
94	*Mesostigma viride*	7371
95	*Mimulus guttatus*	8708
96	*Musa acuminata*	347
97	*Nicotiana benthamiana*	18037
98	*Nicotiana langsdorffii x Nicotiana sanderae*	6791
99	*Nicotiana sylvestris*	7612
100	*Nicotiana tabacum*	89461

List of plant species and their EST sequences obtained from PlantGDB (November, 2008) (cont.).

No.	Plant name	Total EST sequences
101	*Nuphar advena*	13789
102	*Ocimum basilicum*	8926
103	*Oryza sativa*	44644
104	*Oryza sativa indica cultivar group*	84544
105	*Oryza sativa japonica cultivar group*	149282
106	*Oryza sativa Indica Group*	85006
107	*Oryza sativa Japonica Group*	146642
108	*Ostreococcus lucimarinus CCE9901*	13940
109	*Panicum virgatum*	29653
110	*Papaver somniferum*	16486
111	*Paullinia cupana var sorbilis*	10672
112	*Persea americana*	10928
113	*Petunia x hybrida*	9884
114	*Phaseolus angustissimus*	3499
115	*Phaseolus coccineus*	8419
116	*Phaseolus vulgaris*	21361
117	*Physcomitrella patens*	51665
118	*Picea abies*	5184
119	*Picea allspecies*	72011
120	*Picea engelmannii x Picea glauca*	13880
121	*Picea engelmannii x Picea sitchensis*	13880
122	*Picea glauca*	53255
123	*Picea sitchensis*	29178
124	*Pinus pinaster*	12901
125	*Pinus taeda*	77540
126	*Poncirus trifoliata*	35137
127	*Populus alba x Populus tremula*	7528
128	*Populus deltoides*	8186
129	*Populus euphratica*	9117
130	*Populus nigra*	32718
131	*Populus tremula*	19633
132	*Populus tremula x Populus alba*	13517
133	*Populus tremula x Populus tremuloides*	35128
134	*Populus tremuloides*	5730
135	*Populus trichocarpa*	29682
136	*Populus trichocarpa x Populus deltoides*	23686
137	*Populus trichocarpa x Populus nigra*	9740
138	*Populus x canadensis*	4940
139	*Prunus armeniaca*	5637
140	*Prunus persica*	25904
141	*Pseudotsuga menziesii var menziesii*	9857
142	*Raphanus raphanistrum subsp landra*	11855
143	*Raphanus raphanistrum subsp maritimus*	17333
144	*Raphanus raphanistrum subsp raphanistrum*	33369
145	*Raphanus sativus*	22587
146	*Raphanus sativus var oleiformis*	12782
147	*Ricinus communis*	12382
148	*Saccharum officinarum*	131381
149	*Salvia miltiorrhiza*	5624
150	*Saruma henryi*	6754

List of plant species and their EST sequences obtained from PlantGDB (November, 2008) (cont.).

No.	Plant name	Total EST sequences
151	*Secale cereale*	5977
152	*Selaginella moellendorffii*	20542
153	*Solanum chacoense*	7110
154	*Solanum habrochaites*	4024
155	*Solanum lycopersicum*	48945
156	*Solanum pennellii*	3718
157	*Solanum tuberosum*	70344
158	*Sorghum bicolor*	44954
159	*Sorghum propinquum*	9061
160	*Tamarix hispida*	8616
161	*Taraxacum officinale*	18333
162	*Theobroma cacao*	3667
163	*Trifolium pratense*	12746
164	*Triphysaria pusilla*	25947
165	*Triphysaria versicolor*	15255
166	*Triticum aestivum163b*	274754
167	*Triticum aestivum163c*	27011
168	*Triticum monococcum*	6987
169	*Triticum turgidum subsp durum*	7615
170	*Vigna unguiculata*	34736
171	*Vitis shuttleworthii*	5905
172	*Vitis vinifera*	47846
173	*Volvox carteri f nagariensis*	20687
174	*Welwitschia mirabilis*	6606
175	*Zamia vazquezii*	7657
176	*Zea mays*	322003
177	*Zingiber officinale*	16495
178	*Zinnia elegans*	16341
Total		5,306,503
