# Supplementary material for: MicroPC (μPC): A comprehensive resource for predicting and comparing plant microRNAs
Source: BMC Genomics. 2009 Aug 7;10:366. doi: 10.1186/1471-2164-10-366 (PMC2907689; doi:10.1186/1471-2164-10-366)
Supplement: Additional file 4 — The miRNA prediction results for the known 936 precursor miRNA sequences from miRBase and the one thousand repeats of 936 random sequences. [file 1471-2164-10-366-S4.pdf]

The miRNA prediction results for the known 936 precursor miRNA sequences from miRBase and the one thousand repeats of 936 random sequences.

Step	Remaining miRNAs	
	Known precursor miRNA sequences from miRBase	Random sequence (1,000 repeats)
1. Raw data (sequences)	936	936
2. Search miRNA homolog	935	0.126
3. Remove protein coding sequences	911	0.126
4. Remove other types of RNA	910	0.126
5. Fold the 2nd structure of miRNAs	761	0.006
