# Supplementary figures and images for: MicroPC (μPC): A comprehensive resource for predicting and comparing plant microRNAs
Source: BMC Genomics. 2009 Aug 7;10:366. doi: 10.1186/1471-2164-10-366 (PMC2907689; doi:10.1186/1471-2164-10-366)

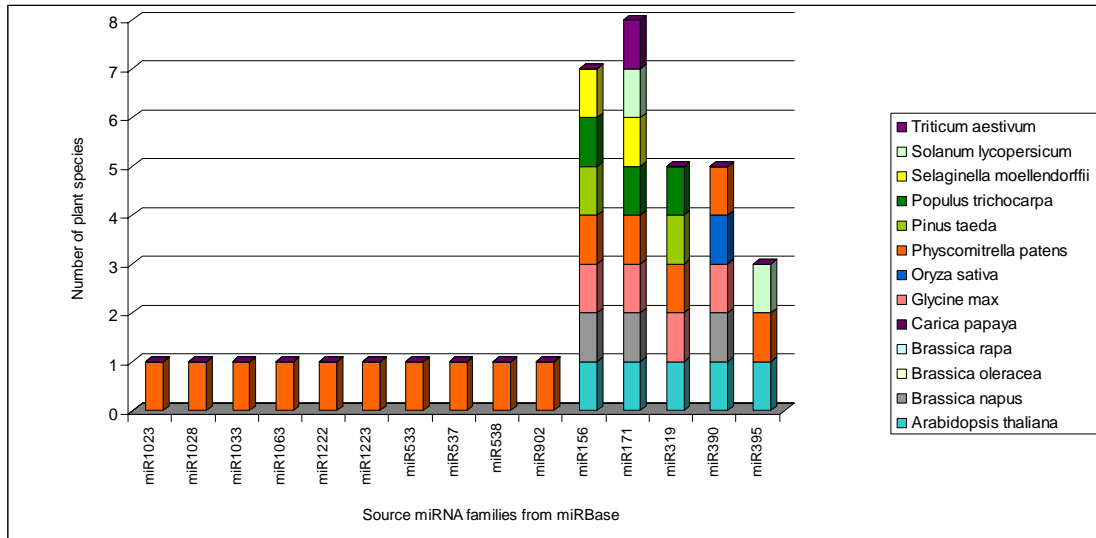

The numbers of plant species of each miRNA family obtained from miRBase.

Supplement: Additional file 5 — The numbers of plant species of each miRNA family obtained from miRBase. [file 1471-2164-10-366-S5.pdf]
