# Supplementary material for: MicroPC (μPC): A comprehensive resource for predicting and comparing plant microRNAs
Source: BMC Genomics. 2009 Aug 7;10:366. doi: 10.1186/1471-2164-10-366 (PMC2907689; doi:10.1186/1471-2164-10-366)
Supplement: Additional file 6 — The numbers of correctly predicted precursor miRNAs from miRBase according to the leave-one-out of source mature miRNA sequences. [file 1471-2164-10-366-S6.pdf]

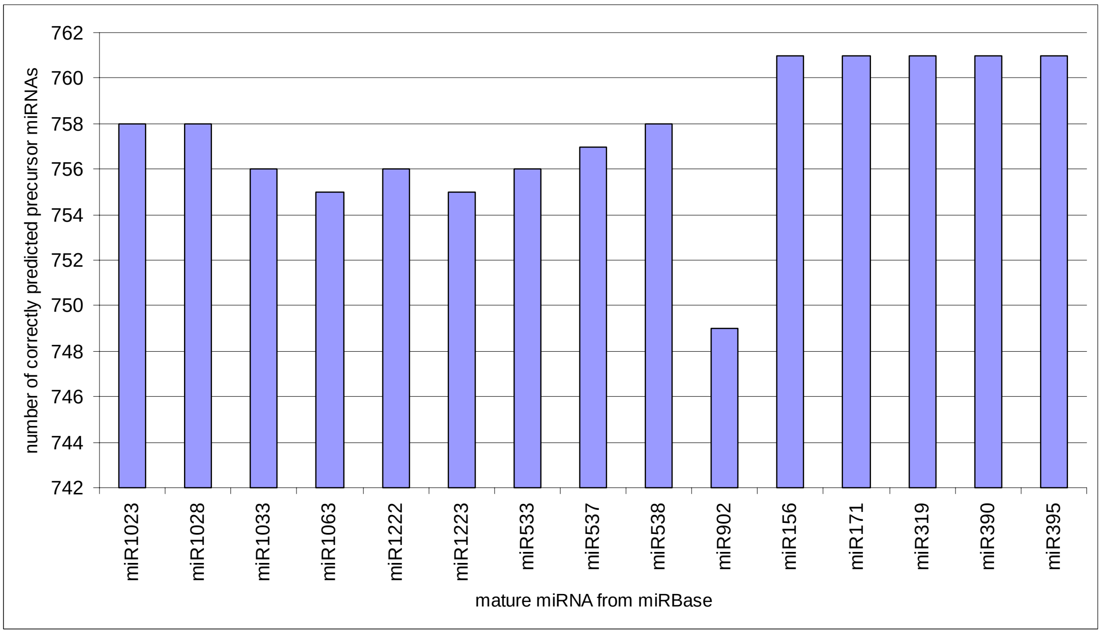

The numbers of correctly predicted precursor miRNAs from miRBase according to the leave-one-out of source mature miRNA sequences.
